# Effects of nutritional supplementation combined with exercise on BMI and lipid profiles in individuals with overweight or obesity: a systematic review and meta-analysis

**DOI:** 10.3389/fnut.2026.1818949

**Published:** 2026-06-15

**Authors:** Hongling Cheng, Chengyu Zhou, Shiwei Song, Bo Wang, Diandong Lang, Peng Sun

**Affiliations:** 1 Department of Police Physical Education and Sports Science, Tianjin Police Institute, Tianjin, China; 2 Strength and Conditioning Training College, Beijing Sports University, Beijing, China; 3 School of Physical Education, Jilin University, Jilin, China; 4 College of Sports and Health, Linyi University, Linyi, China; 5 Physical Education and Health College, AnQing Normal University, Anqing, Anhui Province, China

**Keywords:** blood lipids, BMI, exercise, meta-analysis, nutritional supplementation, obesity

## Abstract

**Background:**

This study systematically evaluated the effects of nutritional supplementation combined with exercise on BMI and lipid profiles in individuals with overweight or obesity.

**Methods:**

A systematic search was conducted in PubMed, Embase, Web of Science, and the Cochrane Library databases from their inception to January 10, 2026, to identify randomized controlled trials (RCTs) comparing “nutritional supplementation combined with exercise” with “placebo.” The primary outcome measure was BMI; secondary outcomes included total cholesterol (TC), triglycerides (TG), low-density lipoprotein cholesterol (LDL-C), and high-density lipoprotein cholesterol (HDL-C). Weight-adjusted mean differences (WMD) and 95% confidence intervals (CI) were calculated using a random-effects model. Data analysis was performed using Stata 15. Study reporting followed the PRISMA statement, and the risk of bias of included randomized controlled trials was assessed using the Cochrane RoB 2.0 tool.

**Results:**

Twelve RCTs involving 315 participants (161 in the intervention group and 154 in the control group) were included. Compared with placebo/control conditions, combined exercise and supplement-based interventions significantly reduced BMI (WMD = −1.72, 95% CI: −2.61 to −0.83; *I*^2^ = 68.7%) and LDL-C (WMD = −15.20, 95% CI: −17.12 to −13.27; *I*^2^ = 0%), increased HDL-C (WMD = 5.74, 95% CI: 3.66–7.83; *I*^2^ = 84.7%), and reduced TG (WMD = −16.54, 95% CI: −25.11 to −7.97; *I*^2^ = 83.1%) and TC (WMD = −15.30, 95% CI: −25.72 to −4.88; *I*^2^ = 92.7%). Subgroup analyses suggested that interventions incorporating aerobic exercise and those lasting 8–12 weeks, particularly 12 weeks, were associated with more favorable outcomes. However, substantial heterogeneity was observed for several lipid outcomes.

**Conclusion:**

Nutritional supplementation combined with exercise may be associated with improvements in BMI and lipid profiles in individuals with overweight or obesity. However, because the included studies varied in supplement type, exercise protocol, intervention duration, and participant characteristics, the findings should be interpreted cautiously. Larger, well-designed randomized trials with standardized intervention protocols and longer follow-up are needed to confirm these effects.

## Background

Obesity and overweight are major public health challenges worldwide and are closely associated with type 2 diabetes, cardiovascular disease, hypertension, and metabolic disorders (1, 2). Among these complications, dyslipidemia is particularly important because elevated total cholesterol (TC), triglycerides (TG), and low-density lipoprotein cholesterol (LDL-C), together with reduced high-density lipoprotein cholesterol (HDL-C) (3), contribute to the development of atherosclerosis and cardiovascular events (4). Therefore, improving body weight status and lipid profiles is an important goal in the management of individuals with overweight or obesity (5, 6).

Lifestyle intervention remains a first-line strategy for obesity management because of its safety, accessibility, and broad applicability (7). Exercise can improve metabolic health by increasing energy expenditure, enhancing insulin sensitivity, promoting fatty-acid oxidation, and regulating lipid metabolism (8). Aerobic exercise is commonly associated with improvements in TG, LDL-C, and HDL-C, whereas resistance training may help preserve or increase lean body mass and support long-term metabolic regulation (9). However, the effects of exercise alone may vary according to adherence, baseline metabolic status, intervention duration, and exercise modality (10).

Nutritional supplementation has also been investigated as an adjunctive strategy for improving obesity-related metabolic outcomes (11, 12). Supplements such as protein, omega-3 fatty acids, dietary fiber, probiotics, vitamins, and plant-derived bioactive compounds may influence satiety, lipid metabolism, inflammation, oxidative stress, and gut microbiota composition (13). When combined with exercise, nutritional supplementation may provide additional metabolic benefits by supporting exercise adaptation, recovery, and lipid regulation. Nevertheless, existing trials have reported inconsistent findings, possibly due to differences in supplement type, dosage, exercise protocol, study population, and intervention duration (14).

Although many randomized controlled trials (15, 16) have examined exercise or nutritional supplementation separately, evidence regarding their combined effects in individuals with overweight or obesity remains fragmented. Therefore, this systematic review and meta-analysis aimed to evaluate the effects of nutritional supplementation combined with exercise on BMI and lipid profiles, including TC, TG, LDL-C, and HDL-C. The findings may help clarify whether combined interventions provide additional metabolic benefits and inform future research on comprehensive obesity management strategies.

## Methods

### Study design and registration

This study was conducted according to the standard procedures for systematic reviews and meta-analyses and reported in accordance with the Preferred Reporting Items for Systematic Reviews and Meta-Analyses (PRISMA) guidelines. The study protocol has been registered with the international systematic review registry PROSPERO (Registration Number: CRD420261308797) to enhance research transparency and reduce selective reporting bias.

### Database sources

The system searched the following electronic databases: PubMed, Embase, Web of Science, and Cochrane Library, from their inception to January 10, 2026. To ensure completeness, reference lists of included studies were also reviewed.

### Search strategy

The search formula combines Medical Subject Headings (MeSH) terms with free-text keywords, primarily comprising the following keyword combinations: (“obesity” OR “overweight”) and (“nutritional supplementation” OR “dietary supplement” OR “protein” OR “omega-3” OR “probiotics” OR “vitamin” OR ‘exercise’ OR “physical activity” OR “aerobic training” OR “resistance training”). Search strategies were adjusted for different databases based on their indexing characteristics. Specific search strategies are detailed in Supplementary Table S1.

### Inclusion and exclusion criteria

#### Inclusion criteria

Studies meeting the following conditions were included:

Study Type: Randomized controlled trials (RCTs).

Study Population: Obese or overweight individuals (defined as BMI ≥ 25 kg/m^2^).

Intervention: Combined exercise and supplement-based interventions. The nutritional interventions included a range of adjunctive supplements, such as spirulina, fisetin, ginger, green tea, vitamin C, omega-3 fatty acids, saffron, astaxanthin, and *Chlorella vulgaris*. Because these interventions differed in composition and potential mechanisms of action, they were treated as a broad category of nutritional adjuncts used alongside exercise rather than as a single uniform intervention. Accordingly, pooled estimates should be interpreted as reflecting the overall effect of exercise combined with heterogeneous nutritional adjuncts, rather than the effect of any specific supplement.

Control: The comparator was defined as the placebo or control group in each included study. Although some trials used different overall grouping schemes, only the data from the corresponding placebo/control arm were extracted for quantitative synthesis.

Outcome Measures: The primary outcome was BMI. Secondary outcomes were TC, TG, LDL, and HDL. Data were extracted for BMI, TC, TG, LDL, and HDL.

Follow-up Duration: Intervention duration ≥4 weeks.

#### Exclusion criteria

Non-randomized studies (observational studies, case–control studies).

Interventions involving only nutritional supplementation or only exercise.

Studies lacking relevant outcome data or with unobtainable data.

Duplicate publications or studies with redundant data reporting (prioritizing the study with the most complete data).

Animal studies or *in vitro* research.

### Literature screening and data extraction

All retrieved literature was first imported into EndNote (Clarivate Analytics) for deduplication. Subsequently, two researchers independently screened the literature according to predefined inclusion and exclusion criteria. The screening process comprised two stages: the first stage involved preliminary screening based on titles and abstracts to exclude studies clearly not meeting inclusion criteria; the second stage involved obtaining full-text articles for eligibility assessment. Disagreements were resolved through discussion; unresolved cases were adjudicated by a third researcher. The literature screening process was visualized using a flowchart following the PRISMA guidelines.

Data extraction was performed independently by two researchers using a standardized, pre-designed data extraction form revised after pilot testing. Extracted data included: (1) Study details: first author, publication year, country; (2) Study characteristics: study design type, sample size; (3) Participant characteristics: age, gender ratio; (4) Intervention measures: type of nutritional supplementation; exercise modality; (5) Specific details of control measures; (6) Outcome measures. For missing or incomplete data, attempts were made to contact original authors via email for Supplementary information. Where required data could not be obtained, its potential impact was assessed in sensitivity analyses. To ensure data accuracy, two researchers cross-checked findings post-extraction. Discrepancies were resolved by re-examining original texts to guarantee final data accuracy and consistency. In addition, baseline metabolic characteristics, including baseline BMI and lipid profile values, were extracted whenever available. Lipid outcomes, including TC, TG, LDL-C, and HDL-C, were uniformly recorded and reported in mg/dL, while BMI was reported in kg/m^2^. If baseline BMI ranges or lipid values were not provided in the original articles, the corresponding data were recorded as “NR” in the characteristics table to avoid selective or unclear reporting.

### Risk of bias

In this study, we used the risk of bias ROB 2.0 tool (17) to assess the quality of the included randomized controlled trials (RCTs). This tool evaluates the risk of bias across five key domains: randomization process, intervention implementation, missing data, measurement bias, and reporting bias. Quality assessments were conducted by two independent researchers, with any disagreements arbitrated by a third researcher. The risk of bias for each study was categorized as “low risk,” “high risk,” or “some concerns.” All assessment results will be included in the appendix, and the potential impact of risk of bias on study outcomes will be discussed.

### Data analysis

All statistical analyses in this study were performed using Stata 15.0 software (Stata Corp, College Station, TX, United States). First, the Cochran Q test and I^2^ statistic were used to assess heterogeneity among included studies. I^2^ values of 0, 25, 50, and 75% indicated no heterogeneity, low heterogeneity, moderate heterogeneity, and high heterogeneity, respectively. Statistically significant heterogeneity was considered present when I^2^ ≥ 50%, and sensitivity analyses were conducted to explore potential sources of heterogeneity. Given potential differences among studies in population characteristics, types of nutritional supplements, exercise modalities, and intervention duration, all outcome measures were analyzed using a random-effects model to obtain more conservative and robust effect estimates. This approach was chosen in part because the included nutritional supplements were mechanistically heterogeneous, even though they were pooled under a broader adjunctive nutritional intervention framework. Continuous variables (BMI, TC, TG, LDL-C, and HDL-C) were expressed as weighted mean differences (WMD) with 95% confidence intervals (95% CI). Publication bias was visually assessed using funnel plots and quantitatively analyzed via Egger regression tests. If the funnel plot showed marked asymmetry or the Egger test indicated publication bias (*p* < 0.10), the trim-and-fill method was applied to adjust for potential publication bias effects on the overall effect size. All statistical tests were two-sided, with *p* < 0.05 considered statistically significant.

## Results

### Literature screening results

**Figure 1:**
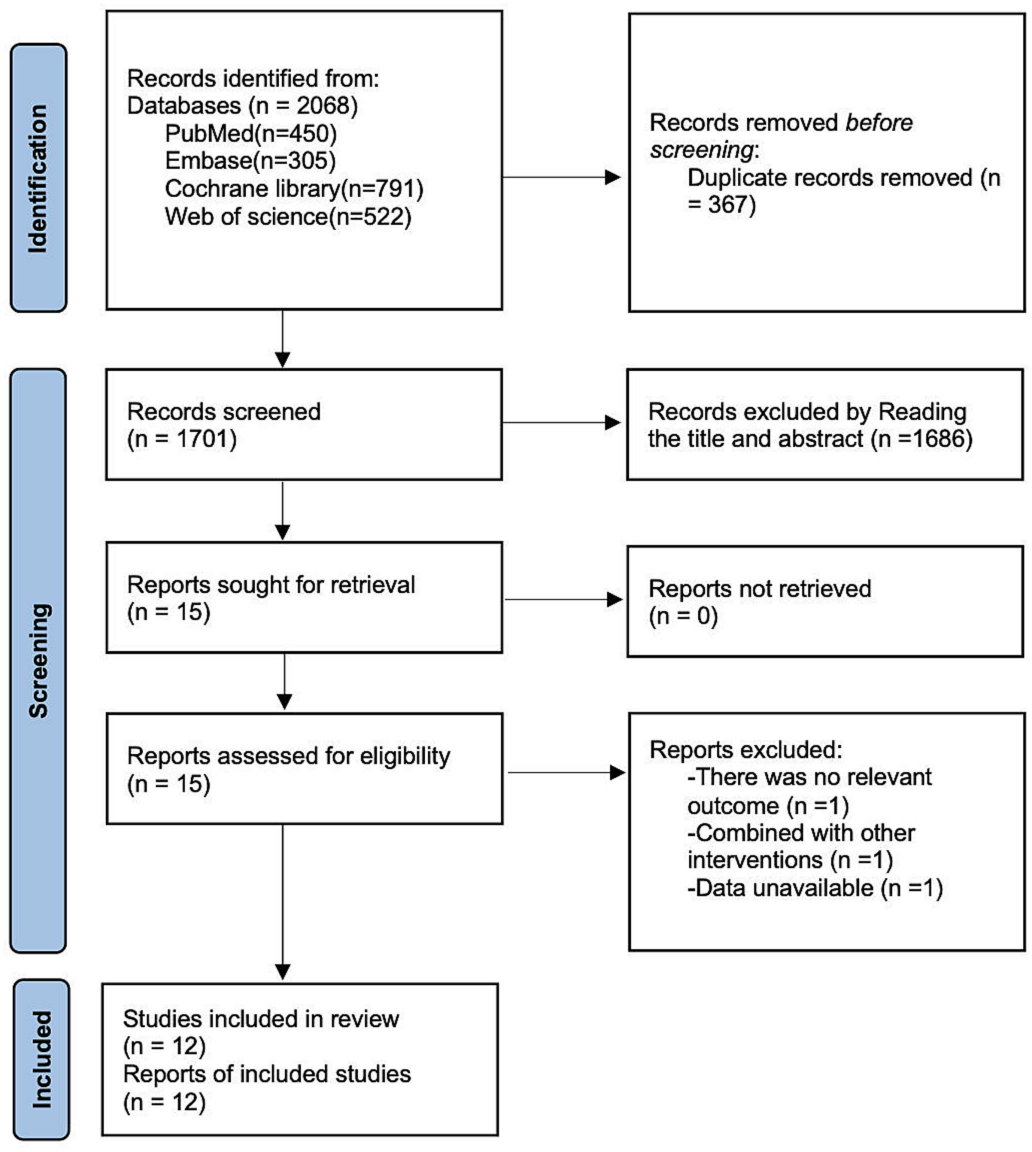
PRISMA flow diagram of study selection. The diagram summarizes literature identification, duplicate removal, title/abstract screening, full-text assessment, exclusion reasons, and final inclusion of randomized controlled trials.

The initial search yielded 2,068 articles, including 450 from PubMed, 305 from Embase, 791 from the Cochrane Library, and 522 from Web of Science. After removing duplicates, 1,701 articles remained. Title and abstract screening excluded 1,686 articles, full-text assessment excluded 15 articles, and 12 randomized controlled trials (18–29) were ultimately included. The literature screening process is detailed in the PRISMA flow diagram (Figure 1).

### Basic characteristics of included studies

This study included 12 randomized controlled trials with a total sample size of 315 participants, comprising 161 in the intervention group (EG) and 154 in the control group (CG). Studies were published between 2011 and 2026, with the majority originating from Iran (*n* = 10), and the remaining from Spain (*n* = 1) and China (*n* = 1). Sample sizes ranged from 16 to 60 participants. The mean age of participants ranged from 15.4 to 58.8 years, encompassing both adolescent populations and middle-aged/elderly obese individuals. Most studies enrolled a single gender group, with only one study using a mixed-gender sample. All studies employed nutritional supplementation combined with exercise intervention. Exercise modalities included aerobic exercise, resistance training, or a combination of both, with intervention durations ranging from 6 to 16 weeks (most commonly 8–12 weeks). Nutritional supplements varied widely, including fisetin, ginger, spirulina, green tea, *Chlorella vulgaris*, vitamin C, omega-3, saffron, and astaxanthin. All studies reported BMI outcomes, with most also reporting lipid profile indicators (LDL-C, HDL-C, TG, and TC). Specific characteristics of the included studies are detailed in Table 1.

**Table 1:** Basic characteristics of the included studies.

Author (year)	Country	Sample size (EG/CG)	Sex (M/F)	Age, years (EG/CG)	Baseline BMI, kg/m^2^ (EG/CG)	Baseline lipid values, mg/dL (EG/CG)	Supplement dosage	Exercise frequency/intensity	Comparator	Outcomes
Alipour et al. (19)	Iran	11/11	22/0	26.64 ± 3.41/26.35 ± 4.21	32.16 ± 0.65/32.58 ± 0.77	EG: LDL-C 139.14 ± 3.08; HDL-C 34.06 ± 2.81; TG 269.92 ± 5.03; TC 248.29 ± 2.15 CG: LDL-C 136.22 ± 3.78; HDL-C 37.84 ± 2.36; TG 269.10 ± 3.71; TC 239.70 ± 4.45	Fisetin, 200 mg/day	Interval resistance-aerobic training; 3 sessions/week for 12 weeks. Resistance: 3 sets × 13 reps at 60% 1RM with active recovery at 20% 1RM; aerobic: 15–25 min at 50–70% HRmax.	Placebo	BMI, LDL-C, HDL-C, TG, TC
Atashak et al. (21)	Iran	8/8	16/0	23.65/25.38	32.5 ± 2.3/32.2 ± 2.3	EG: LDL-C 112.5 ± 31.6; HDL-C 43.2 ± 4.5; TG 145.0 ± 23.0; TC 189.7 ± 41.4 CG: LDL-C 116.9 ± 23.7; HDL-C 42.2 ± 8.5; TG 169.9 ± 38.1; TC 192.5 ± 29.1	Ginger, 1 g/day (four 250 mg capsules/day)	Progressive resistance training; 3 sessions/week for 10 weeks. Intensity progressed from 40 to 50% 1RM to 75–85% 1RM.	Placebo	BMI, LDL-C, HDL-C, TG, TC
Akbarzadeh Khadari et al. (18)	Iran	9/9	0/18	33.2 ± 5.8/36.9 ± 3.5	30.9 ± 2.5/30.1 ± 2.7	NR; lipid outcomes were not reported for this study in the included analysis.	Spirulina platensis, 1 g/day (500 mg twice daily)	Aerobic exercise; 3 sessions/week for 8 weeks, 40–50 min/session. Intensity progressed from 60 to 75% HRmax.	Placebo	BMI
Amozadeh et al. (20)	Iran	13/13	0/26	28.14 ± 7.48/28.13 ± 6.54	33.44 ± 3.78/33.87 ± 4.25	EG: LDL-C 113.62 ± 13.4; HDL-C 39.23 ± 2.61; TG 135.0 ± 15.3; TC 213.69 ± 18.92 CG: LDL-C 109.93 ± 14.1; HDL-C 40.92 ± 3.90; TG 133.3 ± 14.1; TC 188.92 ± 16.70	Green tea, 99 mg/day (33 mg after each main meal)	Aerobic progressive training; 3 sessions/week for 8 weeks, 80–90 min/session. Intensity progressed from 40 to 50% to 70–80% THR.	Control	BMI, LDL-C, HDL-C, TG, TC
Delfan et al. (22)	Iran	11/11	22/0	28.1/28.9	31.9 ± 1.5/32.3 ± 1.1	EG: LDL-C 174.4 ± 16.8; HDL-C 28.8 ± 16.8; TG 261.2 ± 19.1; TC 252.9 ± 15.8 CG: LDL-C 172.0 ± 13.7; HDL-C 30.4 ± 6.4; TG 256.3 ± 15.5; TC 258.2 ± 16.7	*Chlorella vulgaris*, 1800 mg/day	Interval resistance training; 3 sessions/week for 12 weeks, 70 min/session. Resistance: 3 sets at 60% 1RM with active-rest repetitions at 20% 1RM.	Control	BMI, LDL-C, HDL-C, TG, TC
Eskandari et al. (23)	Iran	12/12	24/0	15.41/15.40	32.4 ± 2.2/33.2 ± 2.9	NR; lipid outcomes were not reported for this study in the included analysis.	Dark chocolate, 30 g/day containing 83% cocoa	Jump rope exercise; 5 sessions/week for 6 weeks, 40 min/session. Cadence progressed from 60 to 90 jumps/min.	Control	BMI
Farag et al. (24)	Iran	30/30	24/36	41.2 ± 6.1/42.7 ± 5.3	31.9 ± 5.9/32.5 ± 4.1	EG: LDL-C 137.1 ± 38.4; HDL-C 36.7 ± 10.1; TG 176.3 ± 81.3; TC 179.7 ± 38.6 CG: LDL-C 153.9 ± 38.8; HDL-C 30.8 ± 9.06; TG 152.9 ± 44.6; TC 190.7 ± 38.4	Vitamin C, 500 mg/day	Endurance physical activity for 12 weeks; 30 min/day reported for the intervention arm; detailed intensity NR.	Placebo	BMI, LDL-C, HDL-C, TG, TC
Félix-Soriano et al. (25)	Spain	16/20	0/36	58.13 ± 3.14/58.75 ± 3.39	31.07 ± 1.82/30.25 ± 2.30	EG: LDL-C 164.90 ± 44.04; HDL-C 65.15 ± 11.03; TG 101.34 ± 33.26; TC 250.31 ± 45.89 CG: LDL-C 153.31 ± 32.65; HDL-C 65.74 ± 16.77; TG 92.64 ± 29.47; TC 237.40 ± 30.79	DHA-rich n-3 PUFA, 6 capsules/day providing DHA 1650 mg/day and EPA 150 mg/day	Resistance training; 2 sessions/week for 16 weeks. Intensity progressed from 50 to 80% 1RM; 3–4 series/session with 8–15 repetitions.	Placebo	BMI, LDL-C, HDL-C
Rajabi et al. (26)	Iran	11/11	0/22	54.12 ± 5.63/54.12 ± 5.63	31.02 ± 2.24/35.30 ± 3.63	NR; lipid outcomes were not reported for this study in the included analysis.	Saffron powder, 200 mg/day	Aerobic training; 3 sessions/week for 12 weeks. Main exercise progressed from 20 to 50 min and from 40 to 45% to 70–75% HRmax.	Placebo	BMI
Saeidi et al. (27)	Iran	17/17	34/0	27.6/27.6	33.8 ± 1.2/34.1 ± 2.5	EG: LDL-C 129.1 ± 3.6; HDL-C 37.5 ± 1.4; TG 244.9 ± 5.9; TC 231.5 ± 5.1 CG: LDL-C 127.2 ± 4.4; HDL-C 38.3 ± 1.2; TG 244.1 ± 4.3; TC 228.7 ± 5.2	Astaxanthin, 20 mg/day	High-intensity functional training (CrossFit); 36 sessions over 12 weeks, 60 min/session, 3 sessions/week.	Control	BMI, LDL-C, HDL-C, TG, TC
Sanayei et al. (28)	Iran	12/11	0/23	26.83 ± 5.82/29.27 ± 5.12	30.97 ± 3.26/28.66 ± 2.80	EG: LDL-C 107.08 ± 28.34; HDL-C 47.50 ± 9.08; TG 122.91 ± 41.77; TC 179.16 ± 33.51 CG: LDL-C 91.10 ± 13.15; HDL-C 48.72 ± 8.69; TG 115.27 ± 32.34; TC 162.09 ± 16.86	*Chlorella vulgaris*, 900 mg/day (three 300 mg capsules/day)	HIIT; 3 sessions/week for 8 weeks. Intensity progressed from 50 to 60% HRmax to 90–100% HRmax; each session lasted <1 h.	Control	BMI, LDL-C, HDL-C, TG, TC
Supriya et al. (29)	China	11/11	0/22	28.1/29.1	33.00 ± 1.00/32.77 ± 1.18	EG: LDL-C 176.4 ± 16.83; HDL-C 28.19 ± 5.88; TG 265.8 ± 19.17; TC 258.9 ± 15.77 CG: LDL-C 174.0 ± 13.76; HDL-C 29.76 ± 6.43; TG 260.9 ± 15.51; TC 264.2 ± 16.23	Spirulina, 6 g/day	High-intensity interval training; 3 sessions/week for 12 weeks. Treadmill intervals progressed from 65% VO2peak to 95% VO2peak.	Control	BMI, LDL-C, HDL-C, TG, TC

EG, experimental group; CG, control group; BMI, body mass index; LDL-C, low-density lipoprotein cholesterol; HDL-C, high-density lipoprotein cholesterol; TG, triglycerides; TC, total cholesterol; NR, not reported; HIIT, high-intensity interval training; HRmax, maximum heart rate; THR, target heart rate; 1RM, one-repetition maximum; PUFA, polyunsaturated fatty acids; DHA, docosahexaenoic acid; EPA, eicosapentaenoic acid. Baseline lipid values are reported in mg/dL where available. For multi-arm trials, only the combined exercise plus supplementation arm and the corresponding placebo/control arm used in the meta-analysis are shown.

### Risk of bias results

The quality of 12 randomized controlled trials was assessed using the RoB 2.0 tool (Supplementary Figures S1, S2). Overall, the methodological quality of the included studies was generally favorable, with most trials exhibiting low risk of bias in randomization processes, intervention deviation, completeness of outcome data, and selective reporting of results. Regarding randomization processes, most studies were rated as low risk, with only a few studies raising some concern, primarily due to inadequate reporting of allocation concealment methods. For deviation from the intended intervention, most studies were low risk, while a few were rated as “some concern” due to insufficient descriptions of blinding. Concerning missing outcome data, most studies demonstrated data completeness. Only a few studies provided insufficient explanations for reasons of loss to follow-up or withdrawal, leading to a rating of “some concern.” Regarding outcome measurement bias, some studies were rated as “some concern” due to unclear descriptions of assessor blinding or measurement method details. For selective reporting bias, most studies reported comprehensively, presenting low risk.

### Meta- analysis results

#### BMI

**Figure 2:**
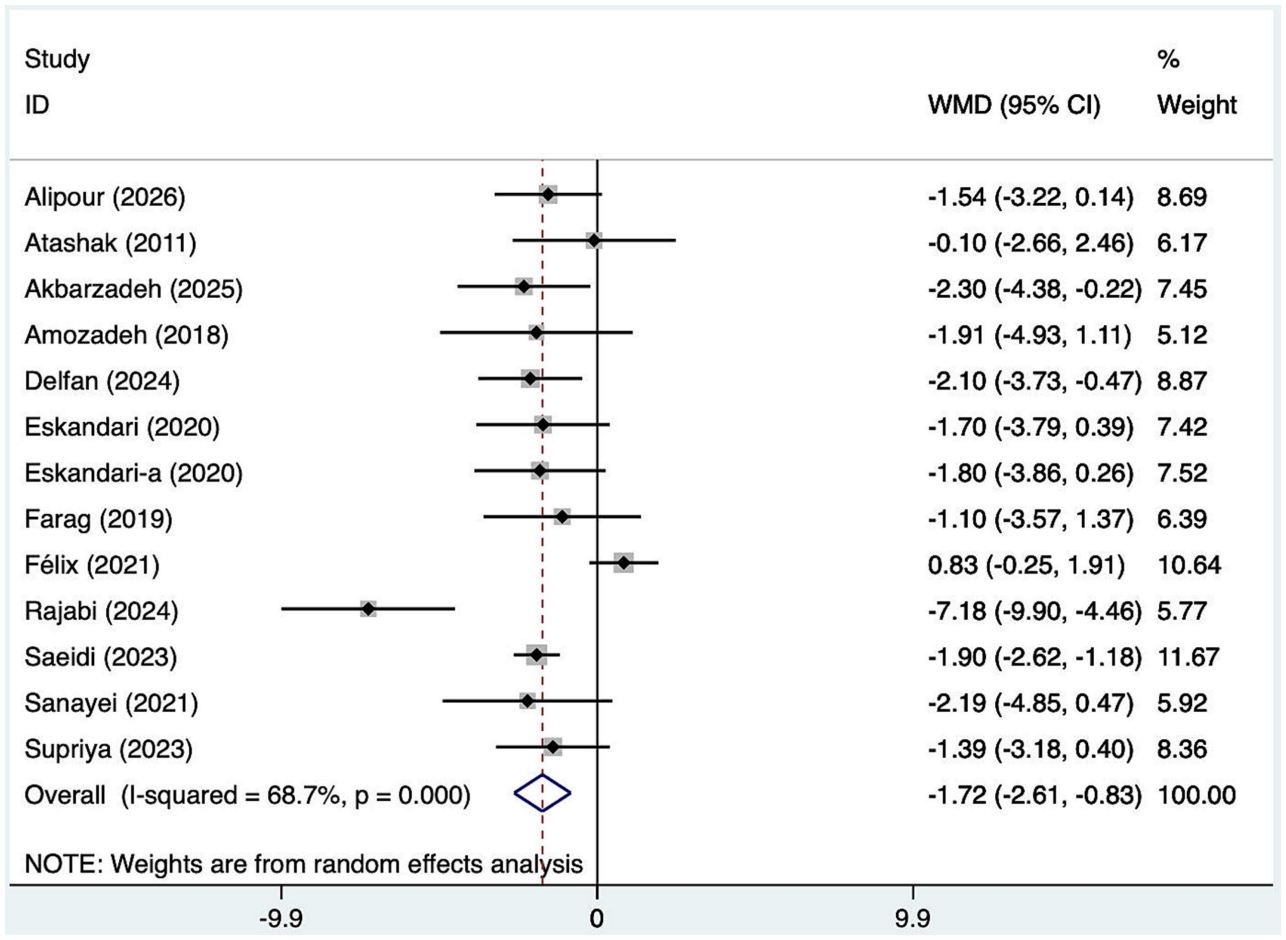
Forest plot of the effect of nutritional supplementation combined with exercise on BMI. Effect estimates are presented as weighted mean differences in kg/m^2^ with 95% confidence intervals.

Twelve articles mentioned BMI. Heterogeneity testing (*I*^2^ = 68.7%, *p* = 0.001), and pooled results (Figure 2) suggest exercise combined with supplements reduces BMI in individuals with overweight or obesity [WMD = −1.72 kg/m^2^, 95% CI (−2.61, −0.83)]. Given significant heterogeneity, sensitivity analysis (Supplementary Figure S3) indicates stable results unaffected by individual studies.

#### LDL

**Figure 3:**
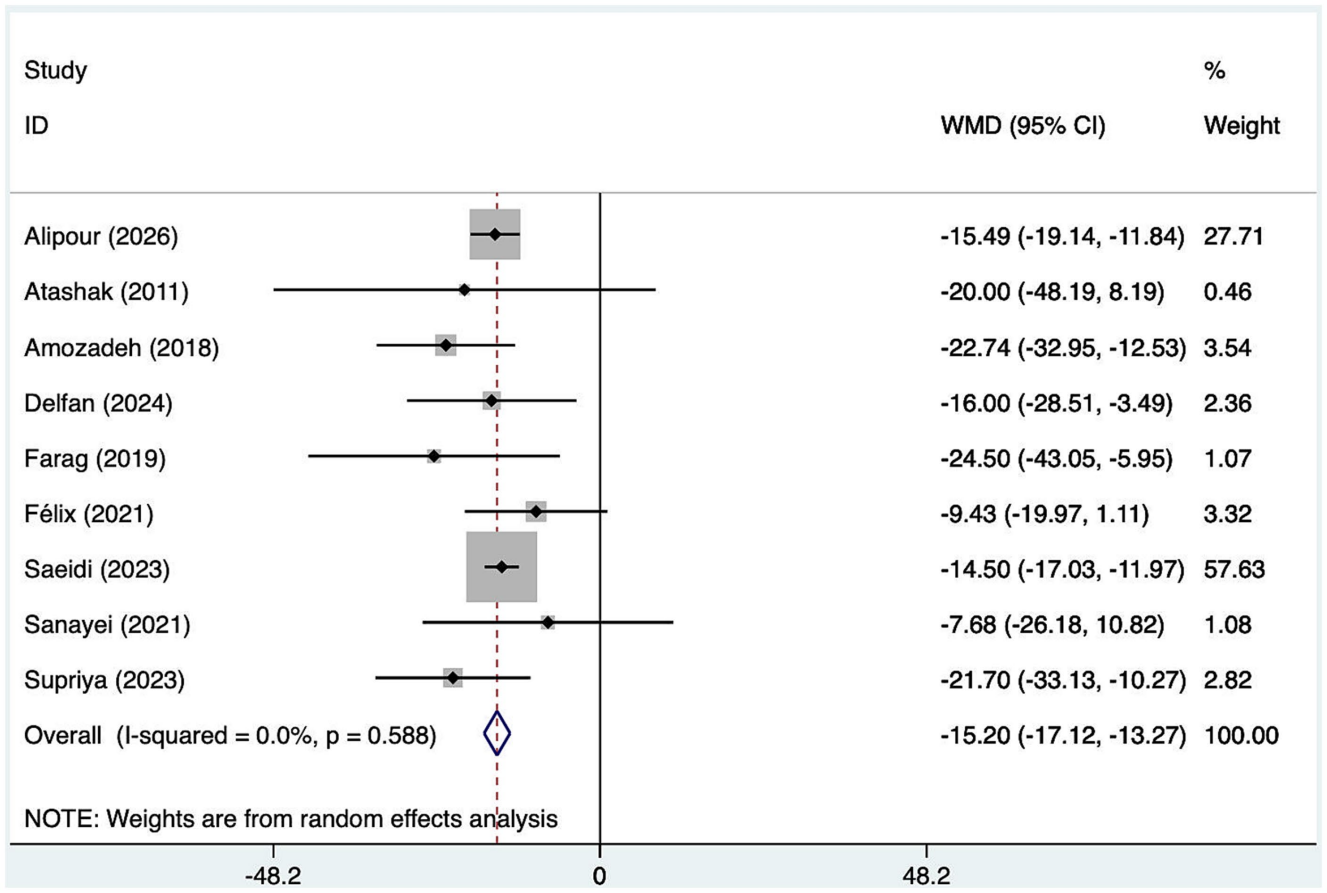
Forest plot of the effect of nutritional supplementation combined with exercise on LDL-C. Effect estimates are presented as weighted mean differences in mg/dL with 95% confidence intervals.

Nine articles mentioned LDL. Heterogeneity testing (*I*^2^ = 0%, *p* = 0.588), and pooled results (Figure 3) suggest exercise combined with supplements reduces LDL in individuals with overweight or obesity [WMD = −15.2 mg/dL, 95% CI (−17.12, −13.27)].

#### HDL

**Figure 4:**
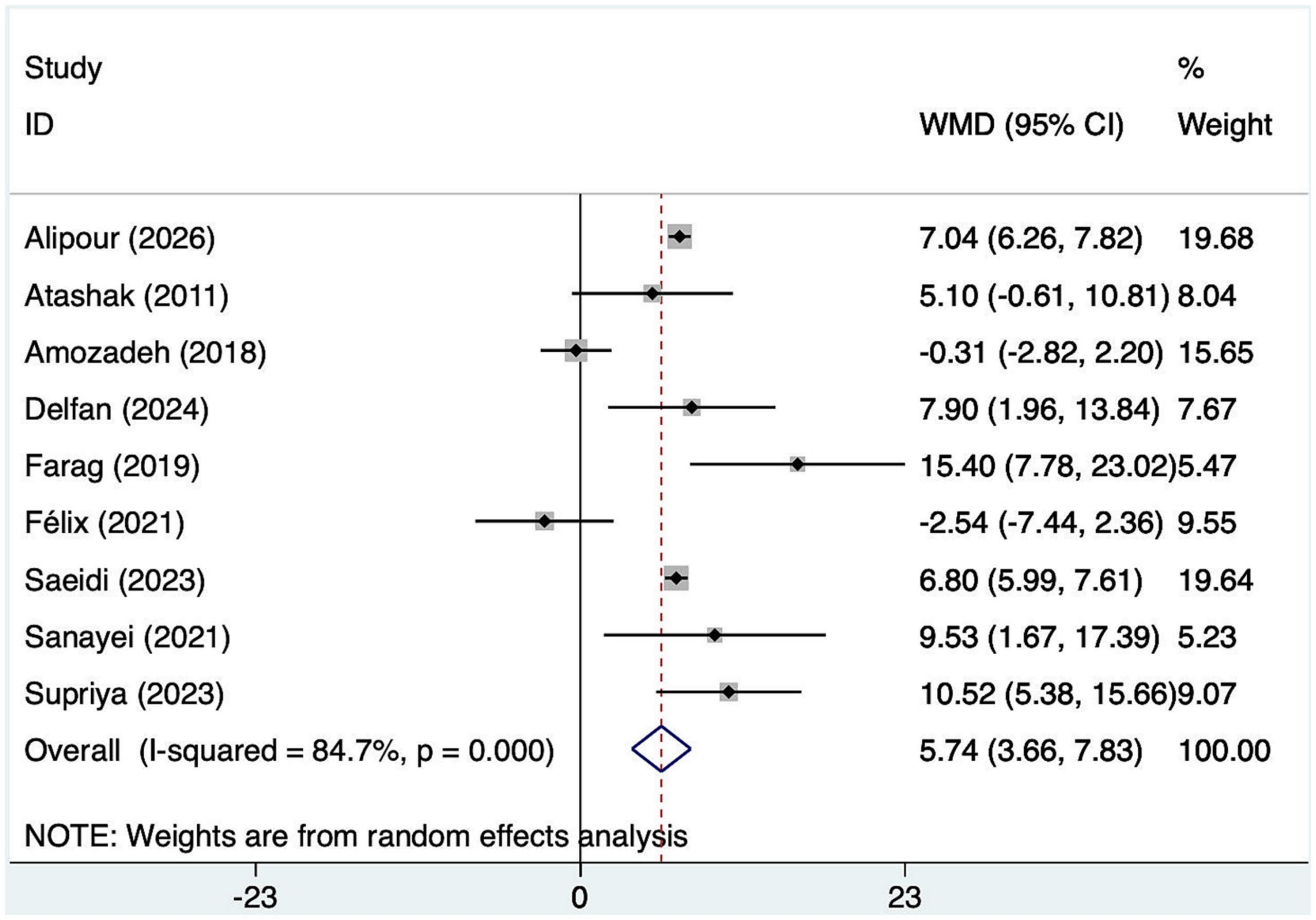
Forest plot of the effect of nutritional supplementation combined with exercise on HDL-C. Effect estimates are presented as weighted mean differences in mg/dL with 95% confidence intervals.

Nine articles mentioned HDL. Heterogeneity testing (*I*^2^ = 84.7%, *p* = 0.001), and pooled results (Figure 4) suggest exercise combined with supplements increase HDL in individuals with overweight or obesity [WMD = 5.74 mg/dL, 95% CI (3.66, 7.83)]. Given significant heterogeneity, sensitivity analysis (Supplementary Figure S4) indicates stable results unaffected by individual studies.

#### TG

**Figure 5:**
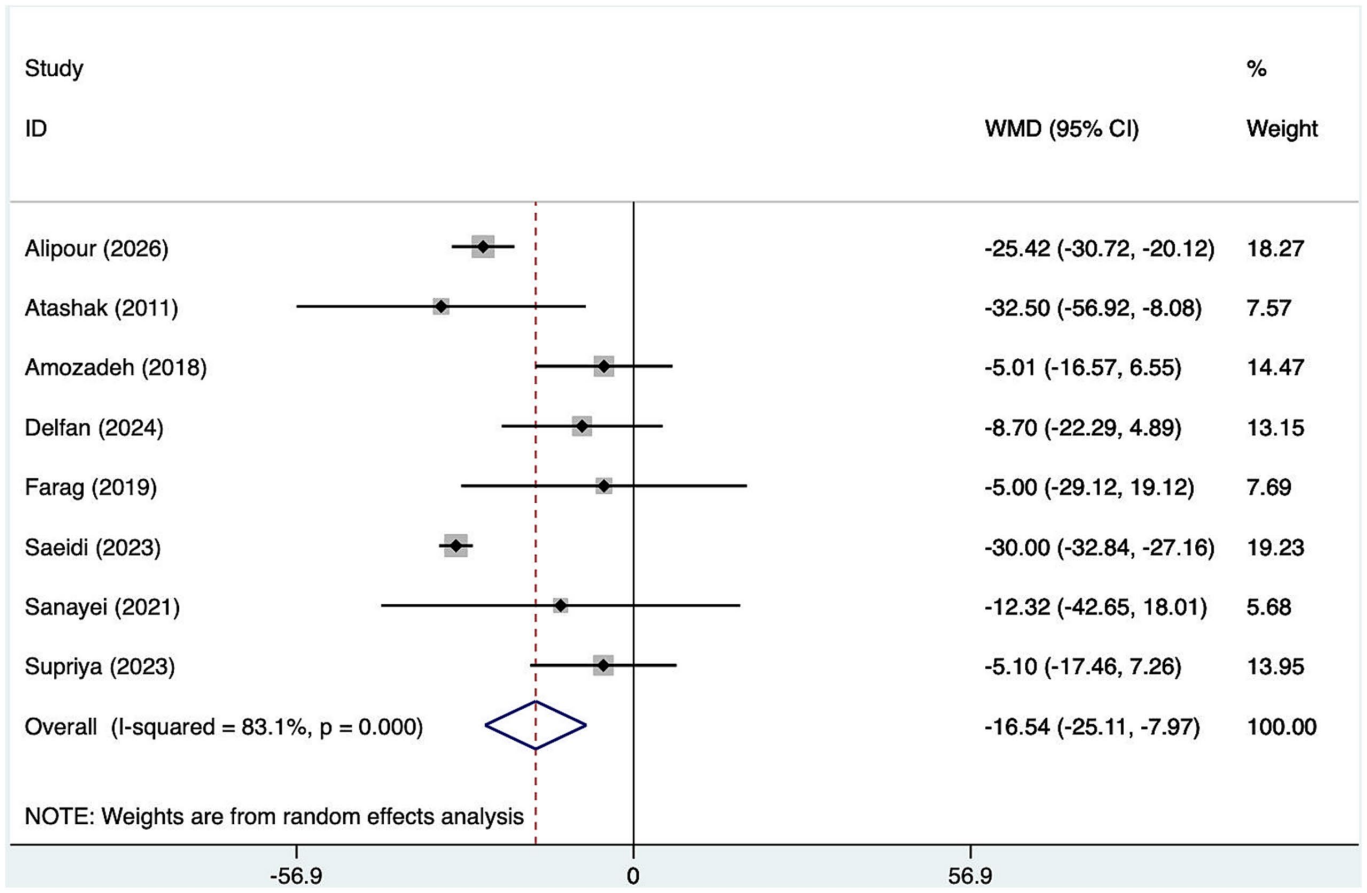
Forest plot of the effect of nutritional supplementation combined with exercise on TG. Effect estimates are presented as weighted mean differences in mg/dL with 95% confidence intervals.

Eight articles mentioned TG. Heterogeneity testing (*I*^2^ = 83.1%, *p* = 0.001), and pooled results (Figure 5) suggest exercise combined with supplements reduces TG in individuals with overweight or obesity [WMD = −16.54 mg/dL, 95% CI (−25.11, −7.97)]. Given significant heterogeneity, sensitivity analysis (Supplementary Figure S5) indicates stable results unaffected by individual studies.

#### TC

**Figure 6:**
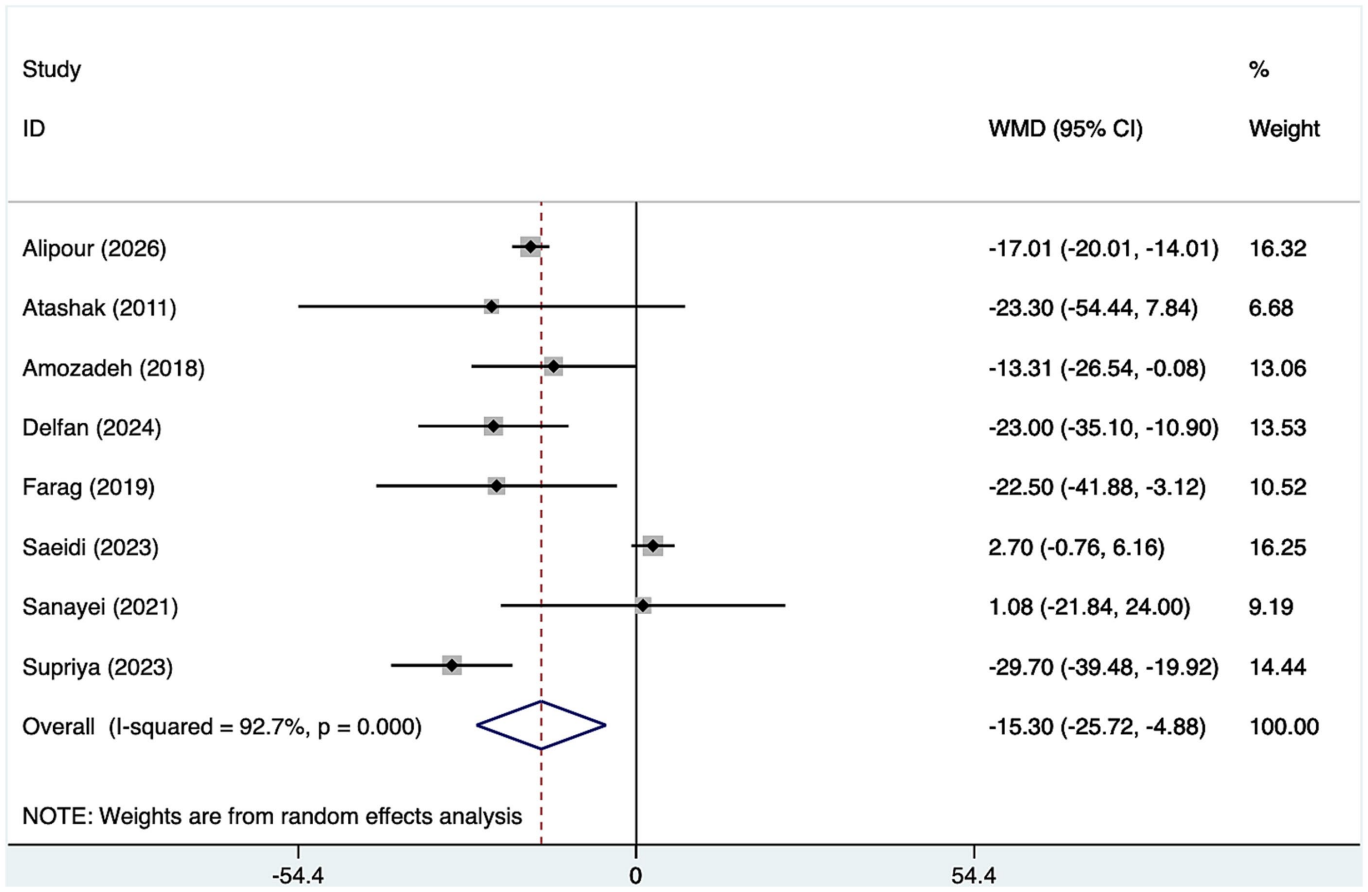
Forest plot of the effect of nutritional supplementation combined with exercise on TC. Effect estimates are presented as weighted mean differences in mg/dL with 95% confidence intervals.

Eight articles mentioned TC. Heterogeneity testing (*I*^2^ = 92.7%, *p* = 0.001), and pooled results (Figure 6) suggest exercise combined with supplements reduces TC in individuals with overweight or obesity [WMD = −15.30 mg/dL, 95% CI (−25.72, −4.88)]. Given significant heterogeneity, sensitivity analysis (Supplementary Figure S6) indicates stable results unaffected by individual studies.

### Subgroup analysis

This study conducted subgroup analyses of BMI and lipid indicators (LDL, HDL, TG, TC) based on intervention methods, intervention time, and gender, respectively.

Subgroup analyses (Table 2) showed that, for BMI, aerobic exercise combined with nutritional supplementation (WMD = −2.33 kg/m^2^, 95% CI: −3.31 to −1.35) and resistance plus aerobic training (WMD = −1.83 kg/m^2^, 95% CI: −3.00 to −0.66) significantly reduced BMI, whereas resistance training alone demonstrated a downward trend but did not reach statistical significance. When stratified by intervention duration, significant improvements were observed at 6 weeks (WMD = −1.75 kg/m^2^, 95% CI: −3.22 to −0.28), 8 weeks (WMD = −2.18 kg/m^2^, 95% CI: −3.62 to −0.74), and 12 weeks (WMD = −2.27 kg/m^2^, 95% CI: −3.45 to −1.10). Sex-stratified analyses indicated significant reductions in both males (WMD = −1.78 kg/m^2^, 95% CI: −2.33 to −1.22) and females (WMD = −2.21 kg/m^2^, 95% CI: −4.36 to −0.06); however, the confidence interval in females approached zero, suggesting relatively weaker stability of the effect.

**Table 2:** Subgroup analysis results.

Outcomes	Group	Subgroup	Number of studies	Heterogeneity (%)	WMD95%CI
BMI	Intervention	Resistance aerobic training	2	0	−1.83 (−3.00, −0.66)
		Resistance training	3	7.4	0.38 (−0.63, 1.40)
		Aerobic exercise	8	51.8	−2.33 (−3.31, −1.35)
	Time	12 week	6	67.2	−2.27 (−3.45, −1.10)
		10 week	1	NA	−0.10 (−2.66, 2.46)
		8 week	3	0	−2.18 (−3.62, −0.74)
		6 week	2	0	−1.75 (−3.22, −0.28)
		16 week	1	NA	0.83 (−0.25, 1.91)
	Gender	Male	6	0	−1.78(−2.33, −1.22)
		Female	6	85.1	−2.21(−4.36, −0.06)
		Both	1	NA	−1.10(−3.57, 1.37)
LDL	Intervention	Resistance aerobic training	2	0	−15.53(−19.03, −12.03)
		Resistance training	3	5.8	−14.11(−23.39, −4.83)
		Aerobic exercise	4	29.6	−16.59(−21.55, −11.64)
	Time	12 week	5	67.2	−15.18(−17.18, −13.17)
		10 week	1	NA	−20.00(−48.19, 8.19)
		8 week	2	0	−17.27(−31.46, −3.07)
		16 week	1	NA	−9.43(−17.12, 1.11)
	Gender	Male	4	0	−14.88(−16.93, −12.83)
		Female	4	37.7	−16.52(−24.14, −8.89)
		Both	1	NA	−24.50(−43.05, −5.95)
HDL	Intervention	Resistance aerobic training	2	0	7.05(6.28, 7.83)
		Resistance training	3	87.1	5.62(−3.99, 15.23)
		Aerobic exercise	4	90.4	6.07(1.26, 10.88)
	Time	12 week	5	41.2	7.23(6.21, 8.25)
		10 week	1	NA	5.10(−0.61, 10.81)
		8 week	2	81.7	3.88(−5.66, 13.41)
		16 week	1	NA	−2.54(−7.44, 2.36)
	Gender	Male	4	0	6.92(6.36, 7.47)
		Female	4	85.3	3.84(−2.28, 9.97)
		Both	1	NA	15.40(7.78, 23.02)
TG	Intervention	Resistance aerobic training	2	80.2	−18.28(−34.49, −2.07)
		Resistance training	2	59.4	−18.08(−45.63, 8.27)
		Aerobic exercise	4	90.3	−13.79(−31.36, 3.77)
	Time	12 week	5	85.1	−17.95(−27.16, −8.74)
		10 week	1	NA	−32.50(−56.92, −8.08)
		8 week	2	0	−5.94(−16.74, 4.87)
	Gender	Male	4	71.8	−24.99(−32.07, −17.92)
		Female	3	0	−5.57(−13.71, 2.56)
		Both	1	NA	−5.00(−29.12, 19.12)
TC	Intervention	Resistance aerobic training	2	0	−17.36(−20.26, −14.45)
		Resistance training	2	0	−22.72(−39.18, −6.27)
		Aerobic exercise	92.6	90.4	−10.25(−28.78, 8.29)
	Time	12 week	5	95.8	−17.11(−30.06, −4.16)
		10 week	1	NA	−23.30(−54.44, 7.84)
		8 week	2	81.7	−9.28(−12.94, 3.38)
	Gender	Male	4	96.1	−13.34(−28.04, 1.37)
		Female	3	74.7	−16.32(−32.82, 0.20)
		Both	1	NA	−22.50(−41.88, −3.12)

For LDL, aerobic exercise (WMD = −16.59 mg/dL, 95% CI: −21.55 to −11.64), resistance plus aerobic training (WMD = −15.53 mg/dL, 95% CI: −19.03 to −12.03), and resistance training alone (WMD = −14.11 mg/dL, 95% CI: −23.39 to −4.83) all significantly reduced LDL levels. The effect was clear at 12 weeks (WMD = −15.18 mg/dL, 95% CI: −17.18 to −13.17). Both male (WMD = −14.88 mg/dL, 95% CI: −16.93 to −12.83) and female (WMD = −16.52 mg/dL, 95% CI: −24.14 to −8.89) subgroups showed statistically significant improvements.

For HDL, resistance plus aerobic training (WMD = 7.05 mg/dL, 95% CI: 6.28–7.83) and aerobic exercise (WMD = 6.07 mg/dL, 95% CI: 1.26–10.88) significantly increased HDL levels; the 12-week intervention (WMD = 7.23 mg/dL, 95% CI: 6.21–8.25) showed relatively consistent effects. The male subgroup showed significant results (WMD = 6.92 mg/dL, 95% CI: 6.36–7.47), whereas some female or single-modality intervention subgroups showed an increasing trend but did not reach statistical significance, as the confidence intervals crossed zero.

For TG, resistance plus aerobic training (WMD = −18.28 mg/dL, 95% CI: −34.49 to −2.07) and the 12-week intervention (WMD = −17.95 mg/dL, 95% CI: −27.16 to −8.74) significantly reduced TG levels. The improvement was more pronounced in males (WMD = −24.99 mg/dL, 95% CI: −32.07 to −17.92), while the female subgroup showed a downward trend without statistical significance.

For TC, resistance plus aerobic training (WMD = −17.36 mg/dL, 95% CI: −20.26 to −14.45) and resistance training alone (WMD = −22.72 mg/dL, 95% CI: −39.18 to −6.27) significantly reduced total TC. The 12-week intervention also reached statistical significance (WMD = −17.11 mg/dL, 95% CI: −30.06 to −4.16). Some short-term interventions or specific sex-based subgroups showed a decreasing trend, but the differences did not reach statistical significance.

### Publication bias

Publication bias was assessed using funnel plots and Egger’s test. The results suggested that publication bias was less likely for BMI (Egger *p* = 0.412), LDL (*p* = 0.360), HDL (*p* = 0.640), and TC (*p* = 0.549), whereas TG showed potential publication bias (Egger *p* = 0.027). To address this, a trim-and-fill analysis was performed for TG. The adjusted effect size was −15.42 mg/dL (95% CI: −23.05 to −7.79), indicating that the overall conclusion of a significant TG reduction with combined nutritional supplementation and exercise remains robust despite potential publication bias (Supplementary Figure S12).

### Meta-regression analysis

To explore potential sources of heterogeneity observed for TG, TC, and HDL (*I*^2^ = 83.1, 92.7, and 84.7%, respectively), we performed exploratory univariable meta-regression analyses using study-level covariates including publication year, country, intervention type, and intervention duration. The results are summarized in Table 3. For TG, intervention type was significantly associated with effect size (*β* = −0.13, *p* = 0.01), suggesting that differences in exercise modality and supplement type may partly explain the observed heterogeneity. Other covariates (year, country, and intervention duration) were not statistically significant for TG. For TC and HDL, none of the examined covariates showed significant associations with effect size (all *p* > 0.05), indicating that the heterogeneity observed for these outcomes could not be explained by the included study-level characteristics. These results are interpreted cautiously due to the limited number of studies available for each outcome, and further research with larger datasets is needed to confirm these findings.

**Table 3:** Meta-regression of study-level covariates on TG, TC, and HDL.

Outcomes	TG (B, P)	TC (B, P)	HDL (B, P)
Year	0.12, 0.87	0.18, 0.75	0.22, 0.44
Country	0.01, 0.13	−0.24, 0.07	−0.32. 0.52
Intervention	−0.13, 0.01	0.93, 0.18	0.65, 0.86
Time	0.52, 0.18	0.84, 0.11	0.22, 0.62

## Discussion

This systematic review and meta-analysis found that nutritional supplementation combined with exercise was associated with reductions in BMI, LDL-C, TG, and TC, and an increase in HDL-C among individuals with overweight or obesity. These findings suggest that combined lifestyle-related interventions may have favorable effects on weight-related and lipid-related outcomes. However, the results should be interpreted cautiously because the included studies differed substantially in supplement type, exercise modality, intervention duration, and participant characteristics.

The observed effects are broadly consistent with evidence indicating that structured exercise can improve metabolic health through increased energy expenditure, enhanced insulin sensitivity, improved fatty-acid oxidation, and regulation of lipoprotein metabolism. Nutritional supplementation may provide additional benefits depending on the specific nutrient, dosage, and baseline metabolic status. Nevertheless, because the included supplements were diverse, including algae-based products, plant extracts, vitamins, omega-3 fatty acids, and other bioactive compounds, the pooled findings should be interpreted as the average effect of heterogeneous combined interventions rather than evidence supporting one specific supplement–exercise regimen.

BMI is a widely used but relatively crude indicator of body weight status. In this meta-analysis, nutritional supplementation combined with exercise was associated with a statistically significant reduction in BMI. However, this finding should not be interpreted as direct evidence of fat-mass reduction or improved body composition (30). BMI does not distinguish between fat mass, lean mass, and fluid changes, and structured exercise, particularly resistance or combined training, may increase or preserve lean mass while reducing fat mass. Therefore, a modest change in BMI may underestimate or incompletely reflect changes in body composition. Because most included trials did not consistently report fat mass, lean mass, waist circumference, or body-fat percentage, we could not determine whether the observed BMI reduction was mainly driven by fat loss, changes in lean mass, or other factors (31). Future studies should report body-composition outcomes to clarify the clinical meaning of BMI changes. The sex-stratified subgroup findings should be interpreted with particular caution. Although some pooled estimates appeared more pronounced in male participants than in female participants, these comparisons were based largely on between-study subgrouping rather than direct within-study comparisons. In addition, several sex-specific subgroups included only a small number of trials and participants, resulting in limited statistical power and unstable estimates. Therefore, the observed sex-related differences should not be considered evidence of true biological effect modification. They are more appropriately interpreted as exploratory findings that may reflect differences in study design, intervention protocol, supplement type, baseline BMI, baseline lipid profile, adherence, or chance. Future trials should prespecify sex-stratified analyses and report outcomes separately by sex to clarify whether sex modifies the response to combined supplementation and exercise interventions.

LDL is recognized as a key risk factor for atherosclerosis. This study demonstrates that combined intervention can significantly reduce LDL by 15.2 mg/dL. This finding may be clinically relevant, as each 1 mmol/L reduction in LDL (approximately 38.7 mg/dL) markedly lowers the risk of cardiovascular events, and the reduction observed in this study has reached the beneficial range for clinical intervention (32, 33). Subgroup analyses revealed that both aerobic exercise, combined resistance and aerobic training, and resistance training alone significantly lowered LDL, indicating relatively minor differences in LDL-lowering effects between exercise modalities. The clear effects observed after 12 weeks further underscores the importance of sustained mid-term interventions. Significant improvements were demonstrated in both male and female subgroups, with the caveat that most studies were single-sex designs; therefore, these gender comparisons should be interpreted cautiously.

HDL possesses anti-atherosclerotic effects. This study found that combined intervention significantly elevated HDL levels (WMD = 5.74 mg/dL), though with considerable heterogeneity (*I*^2^ = 84.7%). Potential contributing factors include variations in supplement types, differing exercise intensities, and baseline metabolic status disparities. Subgroup analyses revealed that both combined resistance and aerobic training and aerobic exercise alone significantly elevated HDL, with effects remaining stable particularly during the 12-week intervention period. Results were significant in the male subgroup, whereas some female subgroups failed to reach statistical significance. This discrepancy may relate to relatively higher baseline HDL levels in women, smaller sample sizes, or hormonal differences (34). Overall, enhancing HDL may require prolonged and regular exercise stimulation.

The findings of this study indicate that combined intervention significantly reduces TG and TC levels. TG decreased by 16.54 mg/dL and TC by 15.30 mg/dL, suggesting that combined intervention exerts a potential regulatory effect on lipid metabolism (35). Subgroup analysis revealed that combined resistance and aerobic training demonstrated particularly pronounced effects in lowering TG and TC, with greater significance observed during the 12-week intervention period. Male participants exhibited more pronounced improvements in TG reduction, whereas female participants showed a downward trend that was partially non-statistically significant. This discrepancy may relate to differences in fat distribution and hormonal regulatory mechanisms (36, 37). Notably, high heterogeneity (*I*^2^ > 80%) was observed in the TG and TC analyses, indicating substantial inter-study variation. Potential sources include differing nutritional supplement types (protein, omega-3 fatty acids, vitamins, or plant extracts), variations in exercise intensity and frequency, and disparities in baseline BMI and lipid profiles. Although Egger’s test suggested potential publication bias for TG, the trim-and-fill adjusted effect size remained statistically significant, indicating that the observed reduction in TG with combined nutritional supplementation and exercise is likely robust. Nevertheless, the possibility of small-study effects cannot be entirely excluded, and the findings should be interpreted cautiously, especially given the limited number of studies for this outcome.

Although the pooled effect sizes were statistically significant for BMI and several lipid outcomes, their clinical interpretation requires caution. The included studies had small sample sizes, short intervention durations, and heterogeneous intervention protocols. In addition, baseline BMI, baseline lipid levels, dietary intake, medication use, and adherence were incompletely reported across trials. These factors may influence the magnitude of treatment effects and limit the generalizability of the pooled estimates. Thus, the effect estimates should be interpreted as approximate average effects across heterogeneous populations and interventions rather than precise estimates applicable to all individuals with overweight or obesity.

The mechanisms underlying the observed improvements in lipid markers are likely multifactorial. First, part of the benefit may have been mediated by reductions in body weight and BMI, as negative energy balance and decreased adiposity can improve lipid metabolism. Increased energy expenditure during exercise may reduce triglyceride storage, improve insulin sensitivity, and secondarily lower LDL, TG, and TC while increasing HDL. However, these effects may not be explained by weight reduction alone. Exercise itself may exert direct metabolic effects independent of body weight change, including enhanced fatty acid oxidation, increased skeletal muscle lipoprotein lipase activity, improved mitochondrial function, greater glucose utilization, and reduced systemic inflammation (38). These adaptations may promote more efficient triglyceride clearance and favorable lipoprotein remodeling even in the absence of substantial weight loss. In addition, some of the included nutritional adjuncts may have contributed through antioxidant, anti-inflammatory, or lipid-regulatory pathways (39). Nevertheless, because most included studies did not adequately report caloric intake, body composition, or mechanistic biomarkers, it was not possible to determine whether the lipid improvements were primarily driven by weight loss-related energy deficit or by direct effects of exercise and supplementation. Therefore, these mechanistic interpretations should be considered suggestive rather than definitive.

### Clinical significance

The findings of this meta-analysis suggest that combining nutritional supplementation with exercise may support improvements in BMI and lipid-related outcomes in individuals with overweight or obesity. In particular, the reduction in LDL-C showed low heterogeneity, suggesting a relatively consistent effect across the included studies. From a clinical perspective, such combined interventions may be considered as part of a non-pharmacological strategy for metabolic health management. However, given the heterogeneity in supplement types and exercise protocols, these findings should not be interpreted as supporting one specific supplement or exercise regimen. Future trials are needed to determine which combinations are most effective and clinically applicable.

### Strengths and limitations

This review has several strengths. First, it included only randomized controlled trials, which improved the reliability of the pooled evidence. Second, the analysis evaluated both BMI and multiple lipid parameters, providing a broader assessment of metabolic outcomes. Third, subgroup, sensitivity, publication bias, and exploratory meta-regression analyses were performed to examine the robustness of the findings and potential sources of heterogeneity.

Several limitations should also be acknowledged. The total sample size was small, and many subgroup analyses were based on a limited number of studies. Most trials were conducted in Iran, which may restrict the generalizability of the findings. In addition, the included studies used different nutritional supplements, exercise modalities, intervention durations, and participant populations, contributing to clinical heterogeneity. Baseline dietary intake, caloric consumption, medication use, adherence, and body-composition outcomes were also incompletely reported. Therefore, the pooled estimates should be interpreted as approximate average effects across heterogeneous interventions rather than definitive evidence for any specific supplement–exercise combination.

## Conclusion

Nutritional supplementation combined with exercise may improve BMI and lipid profiles in individuals with overweight or obesity, including reductions in BMI, LDL-C, TG, and TC and an increase in HDL-C. However, the evidence remains limited by small sample sizes, short intervention durations, and substantial heterogeneity in supplement types and exercise protocols. These findings should therefore be interpreted cautiously. Future large-scale randomized controlled trials with standardized interventions, detailed dietary and metabolic reporting, and longer follow-up are needed to confirm the clinical value of combined supplementation and exercise strategies.

## Data Availability

The original contributions presented in the study are included in the article/Supplementary material, further inquiries can be directed to the corresponding author.
