# Supplementary material for: Effects of nutritional supplementation combined with exercise on BMI and lipid profiles in individuals with overweight or obesity: a systematic review and meta-analysis
Source: Front Nutr. 2026 Jun 15;13:1818949. doi: 10.3389/fnut.2026.1818949 (PMC13312902; doi:10.3389/fnut.2026.1818949)
Supplement: Supplementary file 1 [file Data_sheet_1.docx]

Table S1: search strategies

(((("Dietary Supplements"[Mesh]) OR (((((((((((((((((((Supplements[Title/Abstract]) OR (Dietary Supplement[Title/Abstract])) OR (Supplements, Dietary[Title/Abstract])) OR (Dietary Supplementations[Title/Abstract])) OR (Supplementations, Dietary[Title/Abstract])) OR (Food Supplementations[Title/Abstract])) OR (Food Supplements[Title/Abstract])) OR (Food Supplement[Title/Abstract])) OR (Supplement, Food[Title/Abstract])) OR (Supplements, Food[Title/Abstract])) OR (Nutraceuticals[Title/Abstract])) OR (Nutraceutical[Title/Abstract])) OR (Nutriceuticals[Title/Abstract])) OR (Nutriceutical[Title/Abstract])) OR (Neutraceuticals[Title/Abstract])) OR (Herbal Supplements[Title/Abstract])) OR (Herbal Supplement[Title/Abstract])) OR (Supplement, Herbal[Title/Abstract])) OR (Supplements, Herbal[Title/Abstract]))) AND (("Exercise"[Mesh]) OR ((((((((((((((((((((((((((((Exercise[Title/Abstract]) OR (Exercises[Title/Abstract])) OR (Exercise, Physical[Title/Abstract])) OR (Exercises, Physical[Title/Abstract])) OR (Physical Exercise[Title/Abstract])) OR (Physical Exercises[Title/Abstract])) OR (Exercise, Isometric[Title/Abstract])) OR (Exercises, Isometric[Title/Abstract])) OR (Isometric Exercises[Title/Abstract])) OR (Isometric Exercise[Title/Abstract])) OR (Exercise, Aerobic[Title/Abstract])) OR (Aerobic Exercise[Title/Abstract])) OR (Aerobic Exercises[Title/Abstract])) OR (Exercises, Aerobic[Title/Abstract])) OR (Exercise Training[Title/Abstract])) OR (Exercise Trainings[Title/Abstract])) OR (Training, Exercise[Title/Abstract])) OR (Trainings, Exercise[Title/Abstract])) OR (Physical Activity[Title/Abstract])) OR (Activities, Physical[Title/Abstract])) OR (Activity, Physical[Title/Abstract])) OR (Physical Activities[Title/Abstract])) OR (Active Breaks[Title/Abstract])) OR (Activity Breaks[Title/Abstract])) OR (Acute Exercise[Title/Abstract])) OR (Acute Exercises[Title/Abstract])) OR (Exercise, Acute[Title/Abstract])) OR (Exercises, Acute[Title/Abstract])))) AND (("Overweight"[Mesh]) OR ((Overweight[Title/Abstract]) OR (Obesity[Title/Abstract])))) AND (randomized controlled trial[Publication Type] OR randomized[Title/Abstract] OR placebo[Title/Abstract])


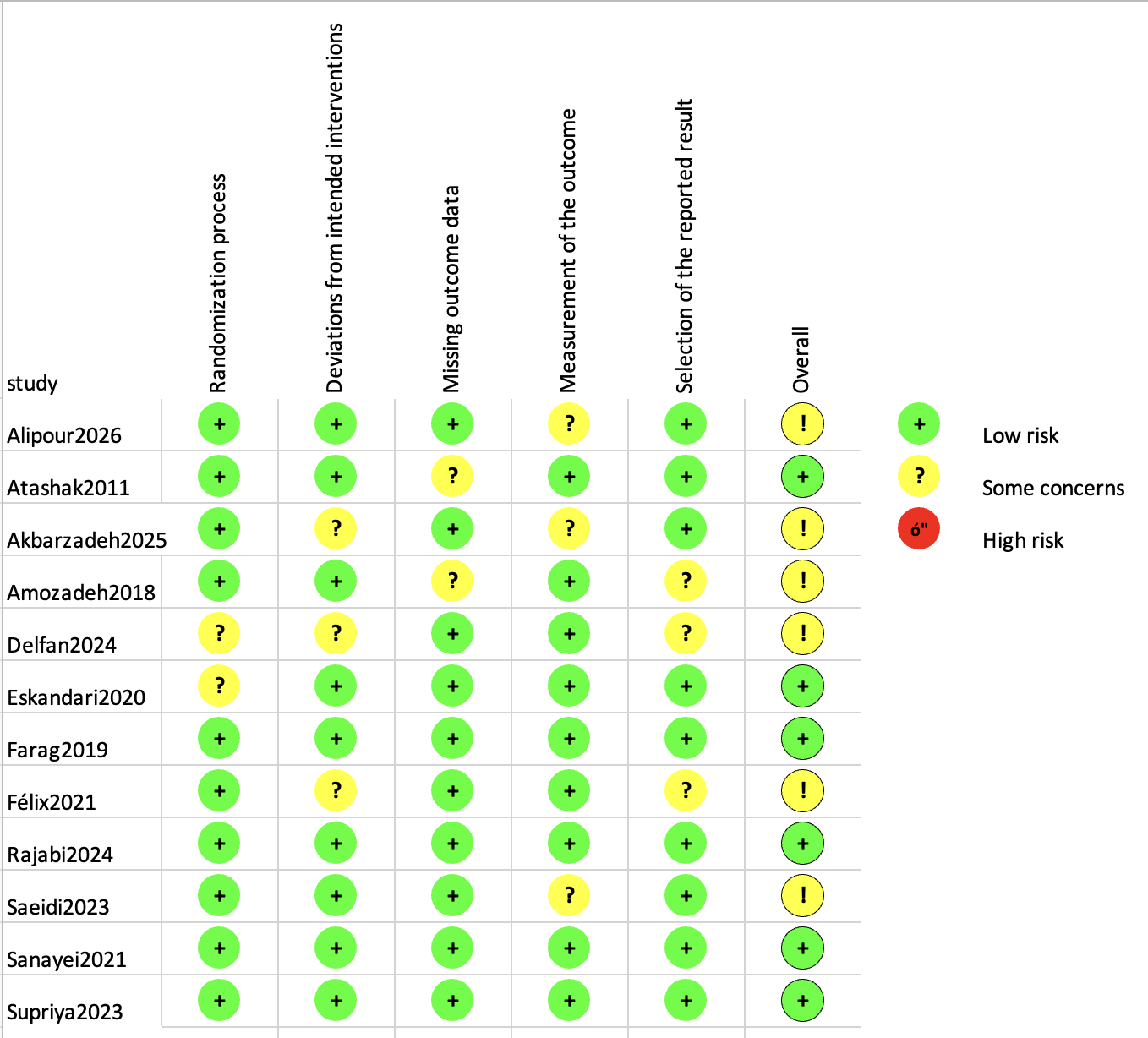


Figure S1 risk bias of summary


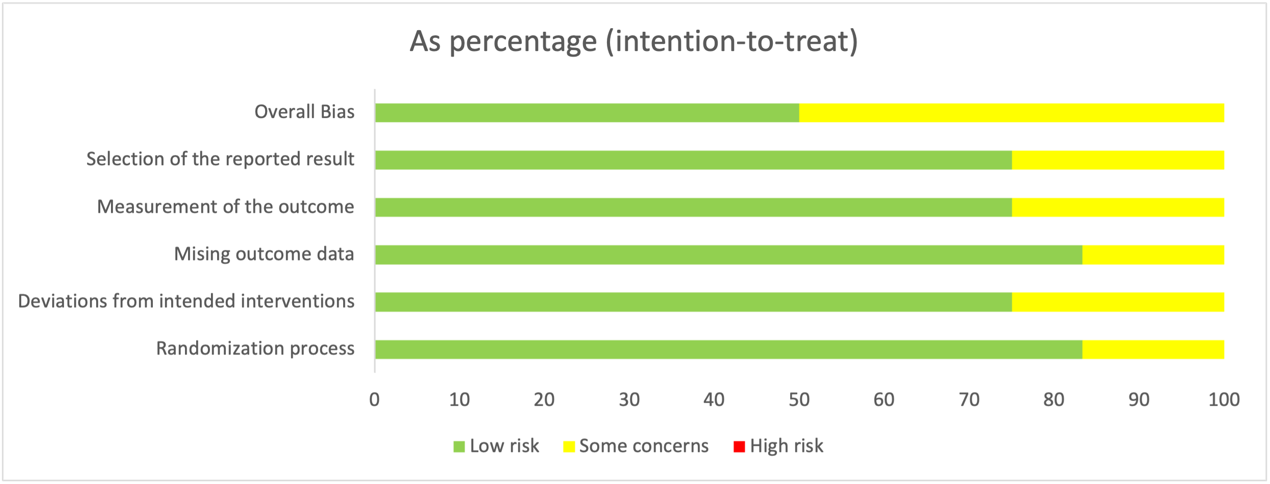


Figure S2 risk bias of graph


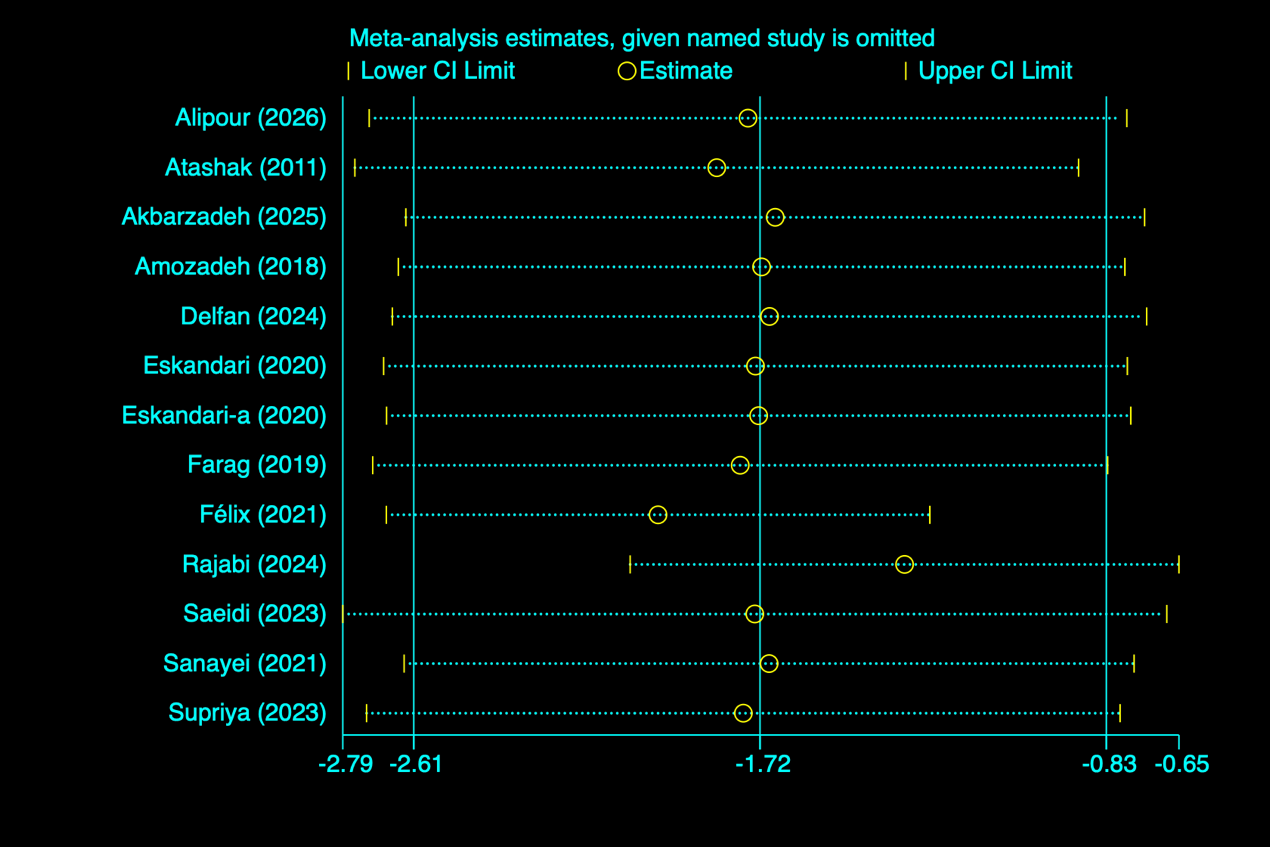


Figure S3 Sensitivity analysis of BMI


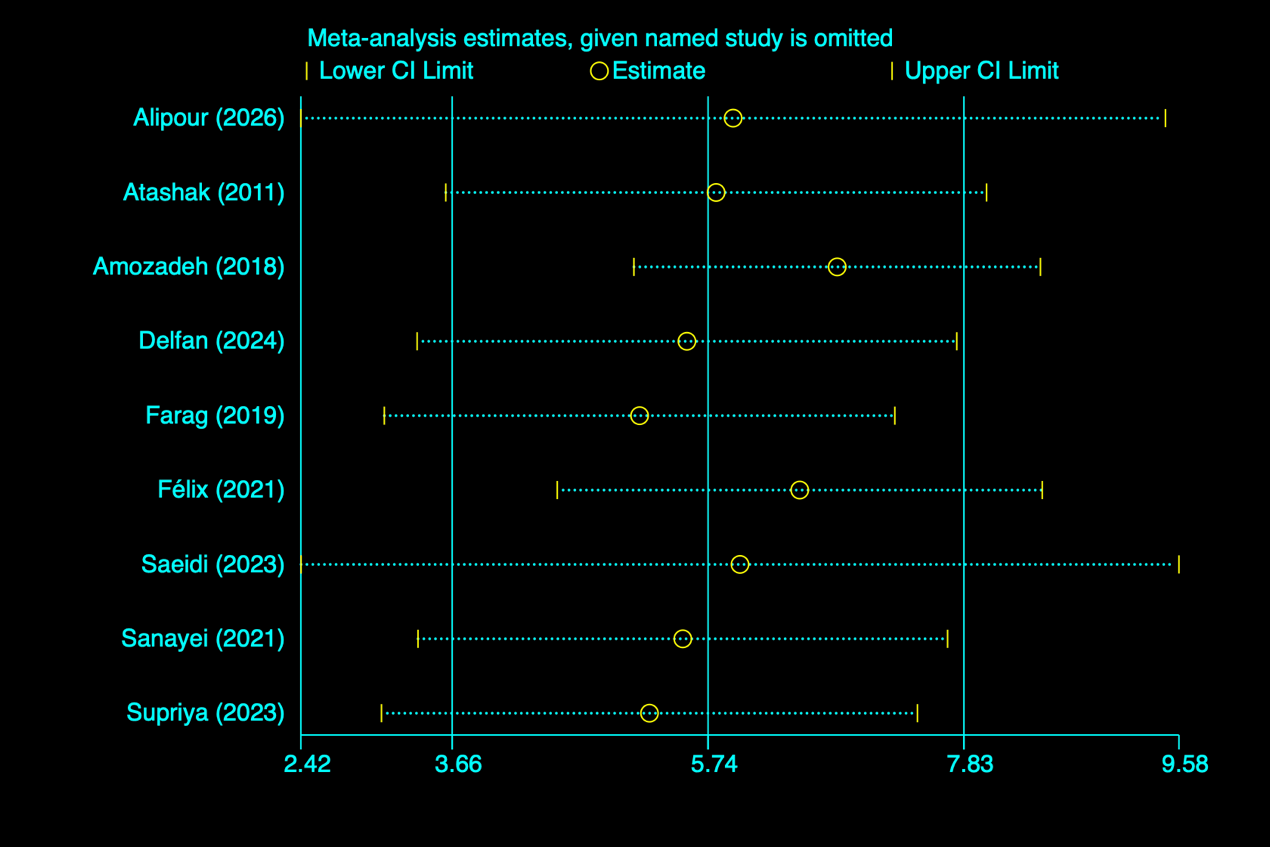


Figure S4 Sensitivity analysis of HDL


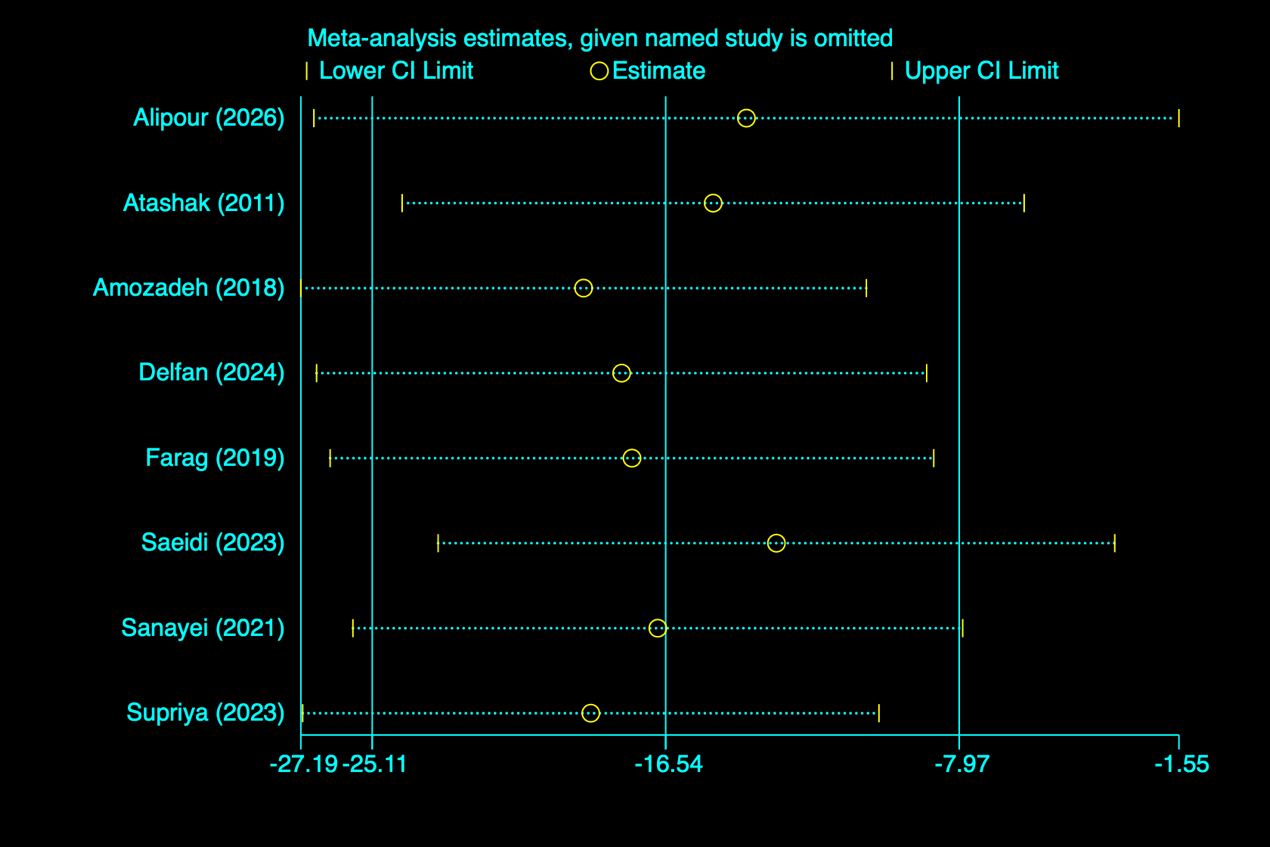


Figure S5 Sensitivity analysis of TG


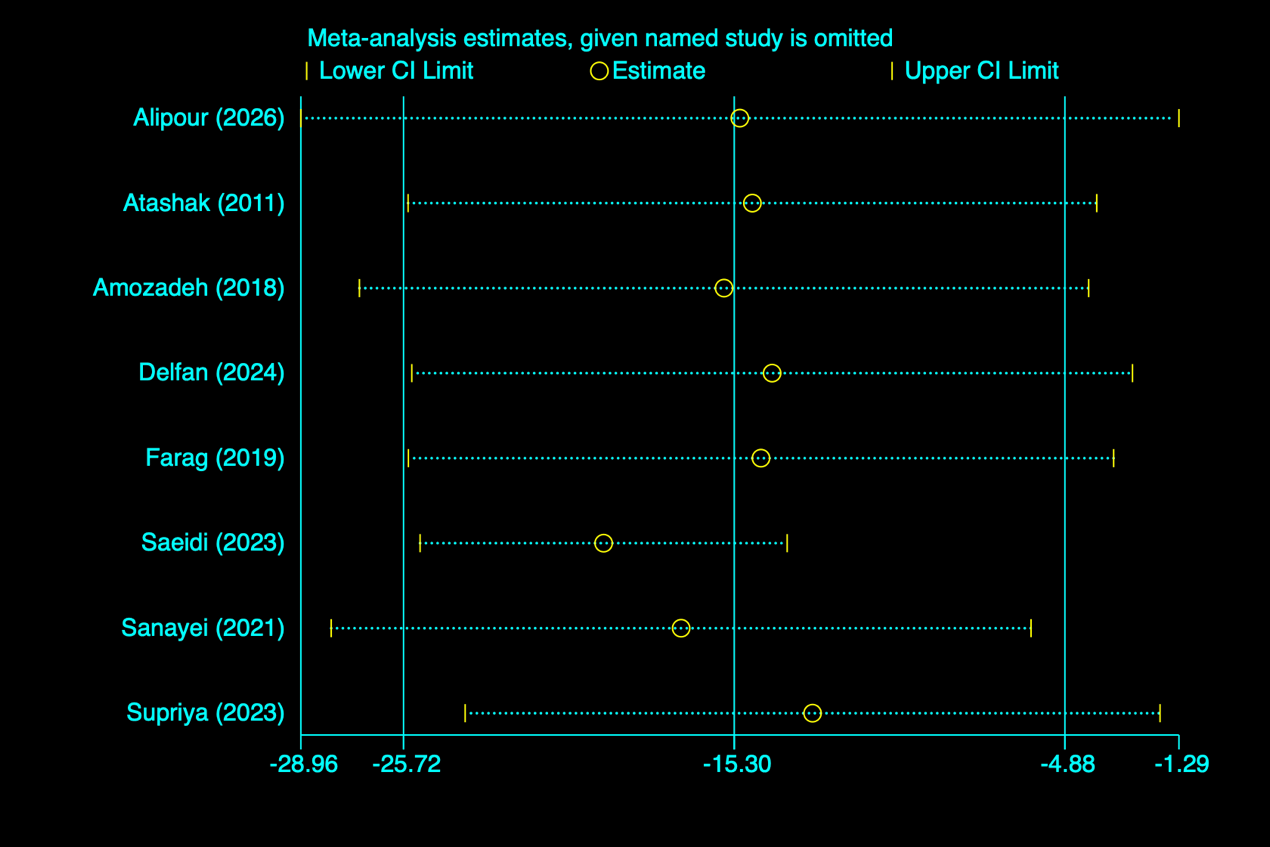


Figure S6 Sensitivity analysis of TC


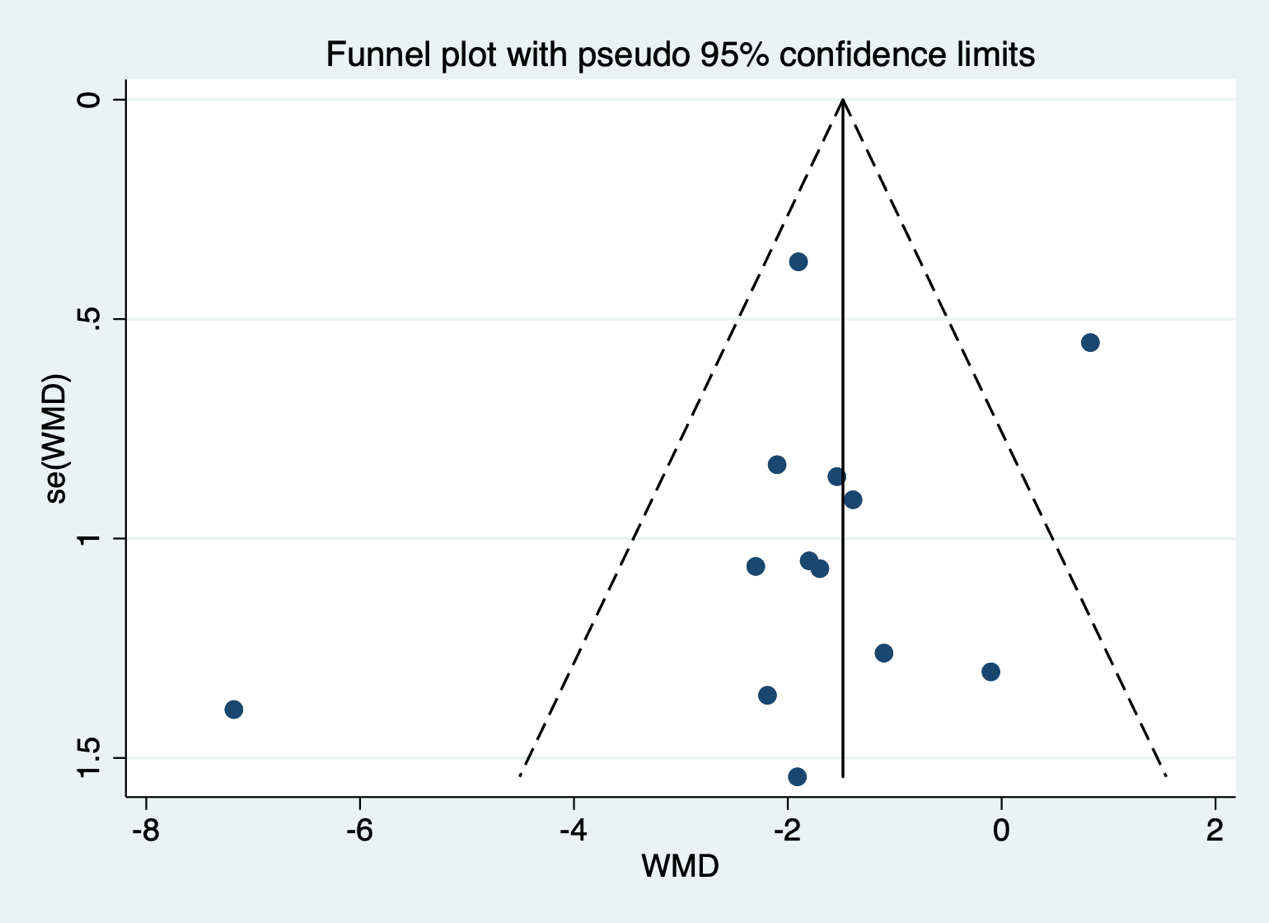


Figure S7 Meta-analysis funnel plot of BMI


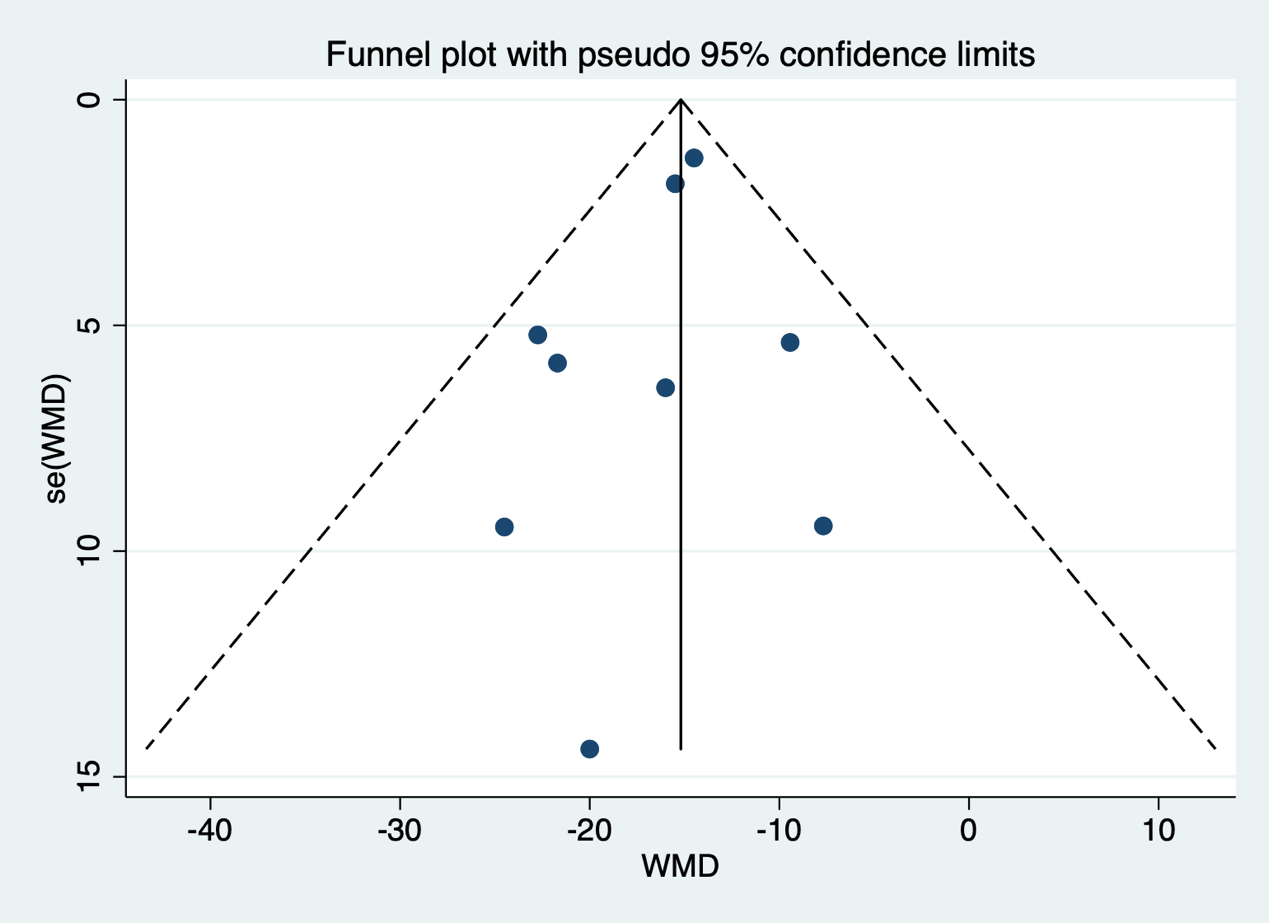


Figure S8 Meta-analysis funnel plot of LDL


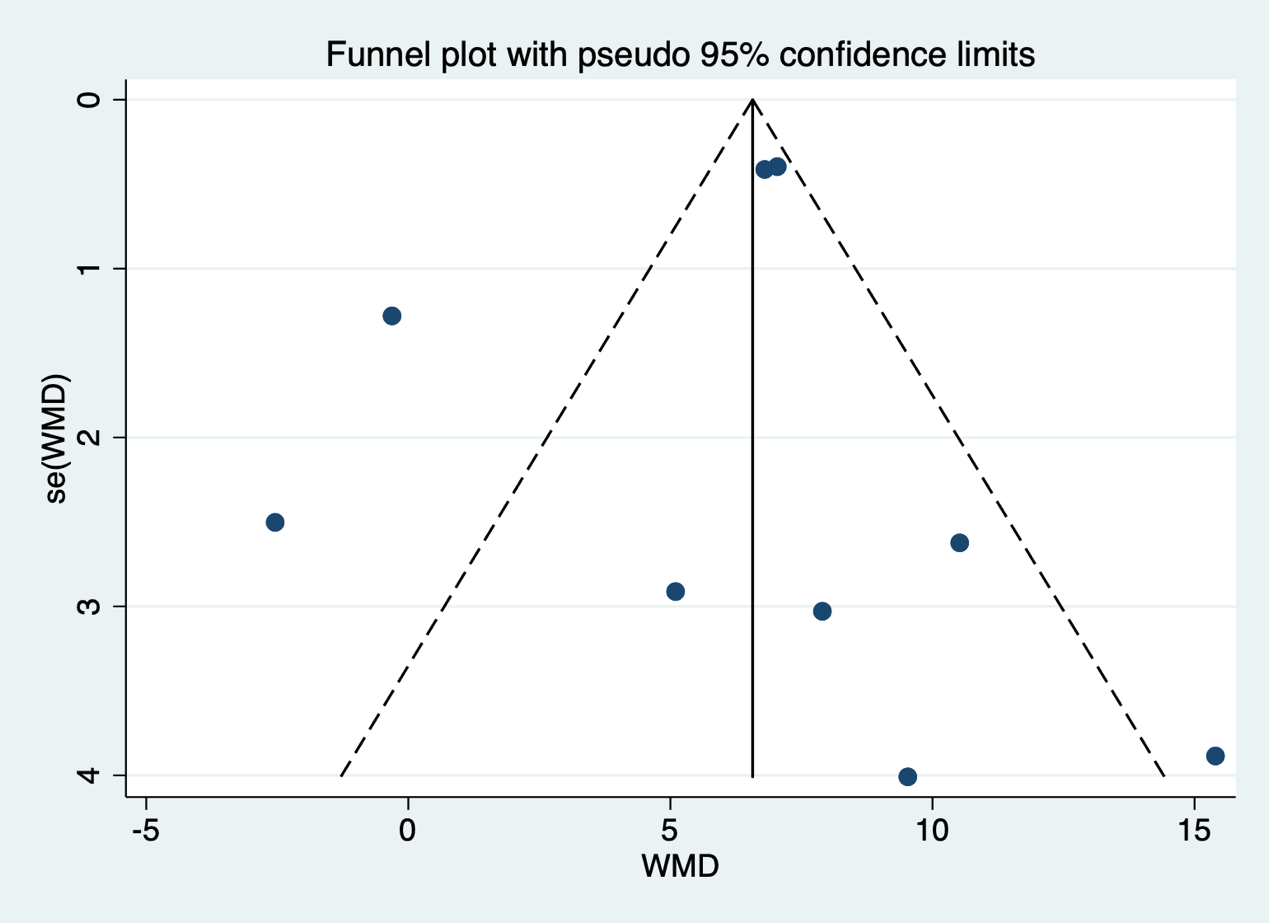


Figure S9 Meta-analysis funnel plot of HDL


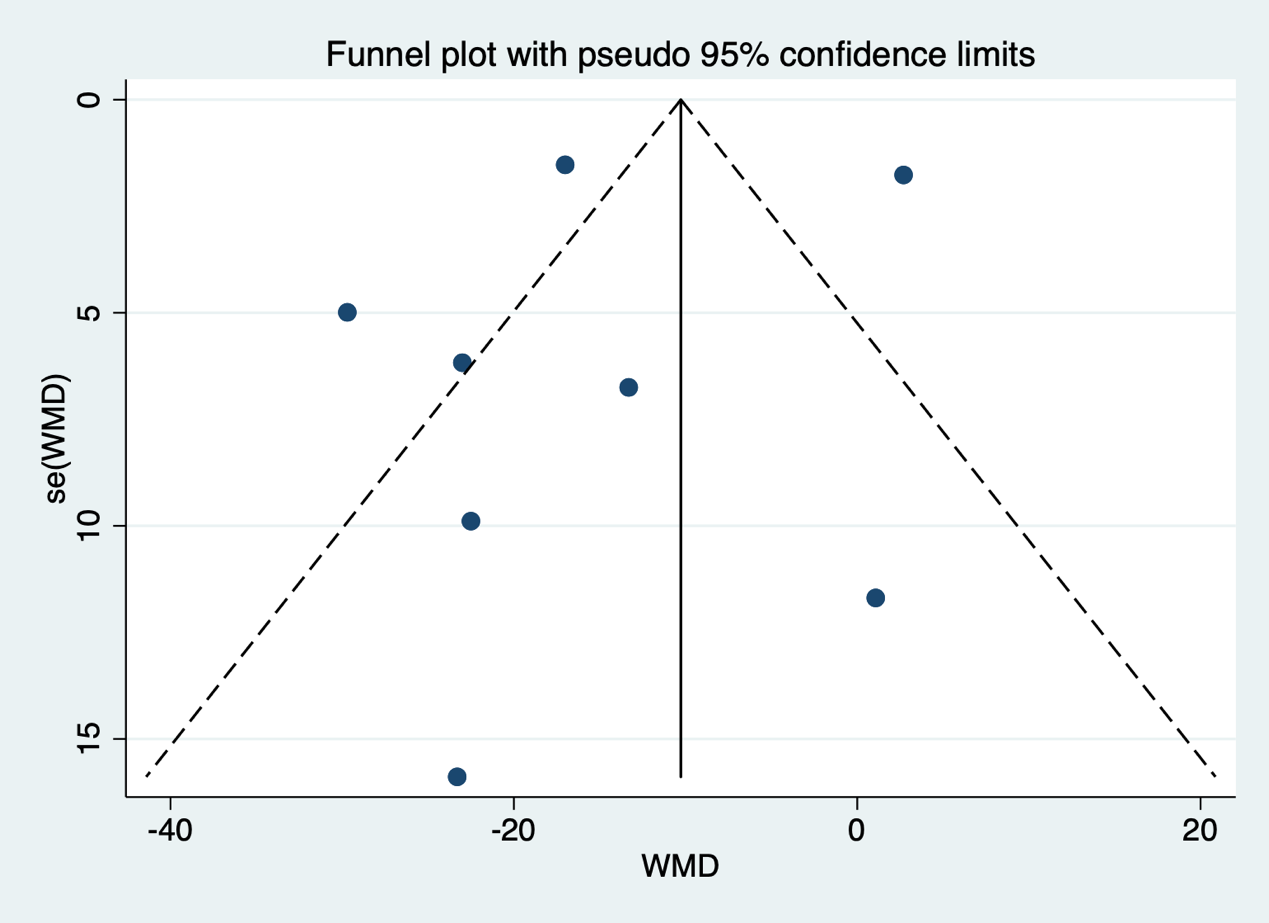


Figure S10 Meta-analysis funnel plot of TC


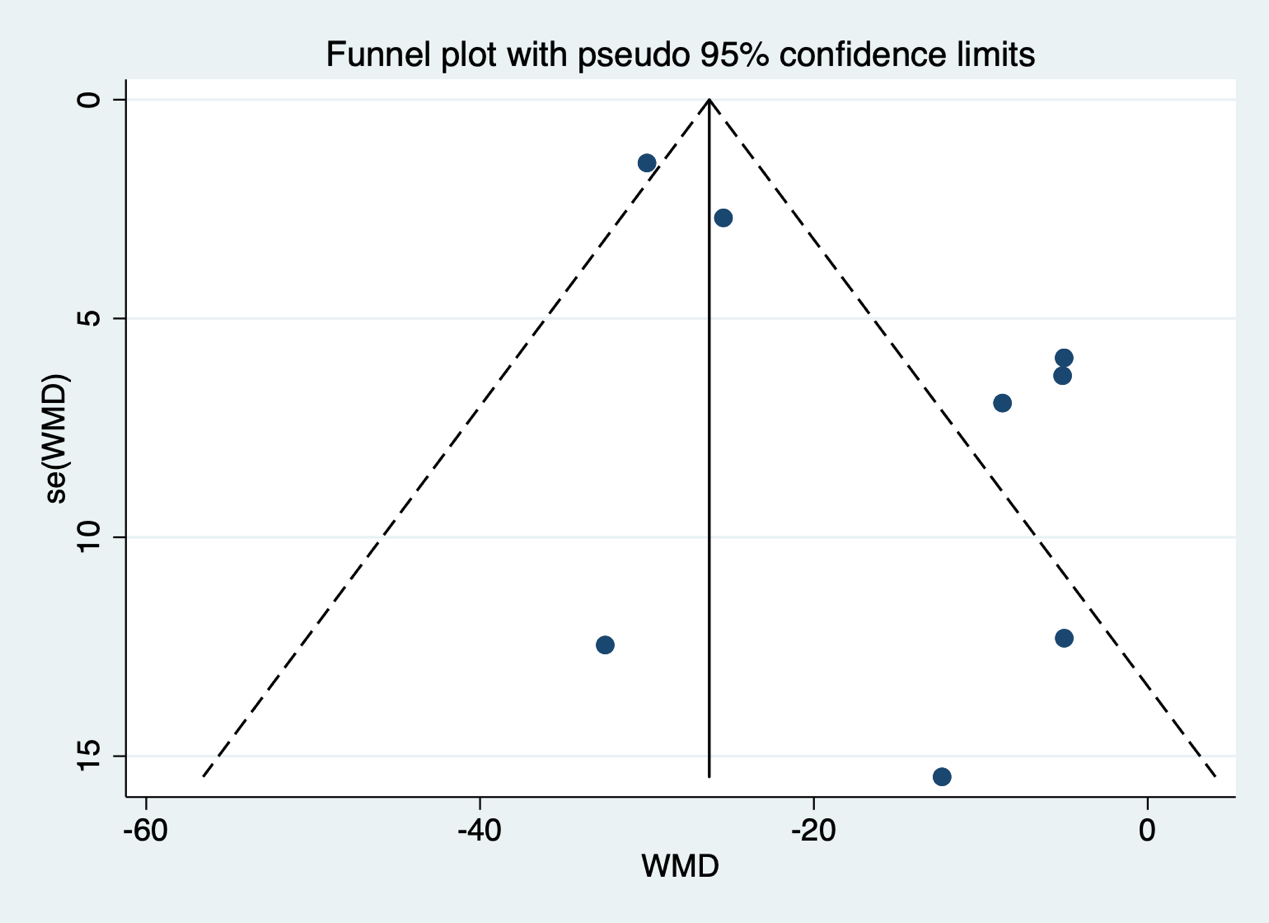


Figure S11 Meta-analysis funnel plot of TG


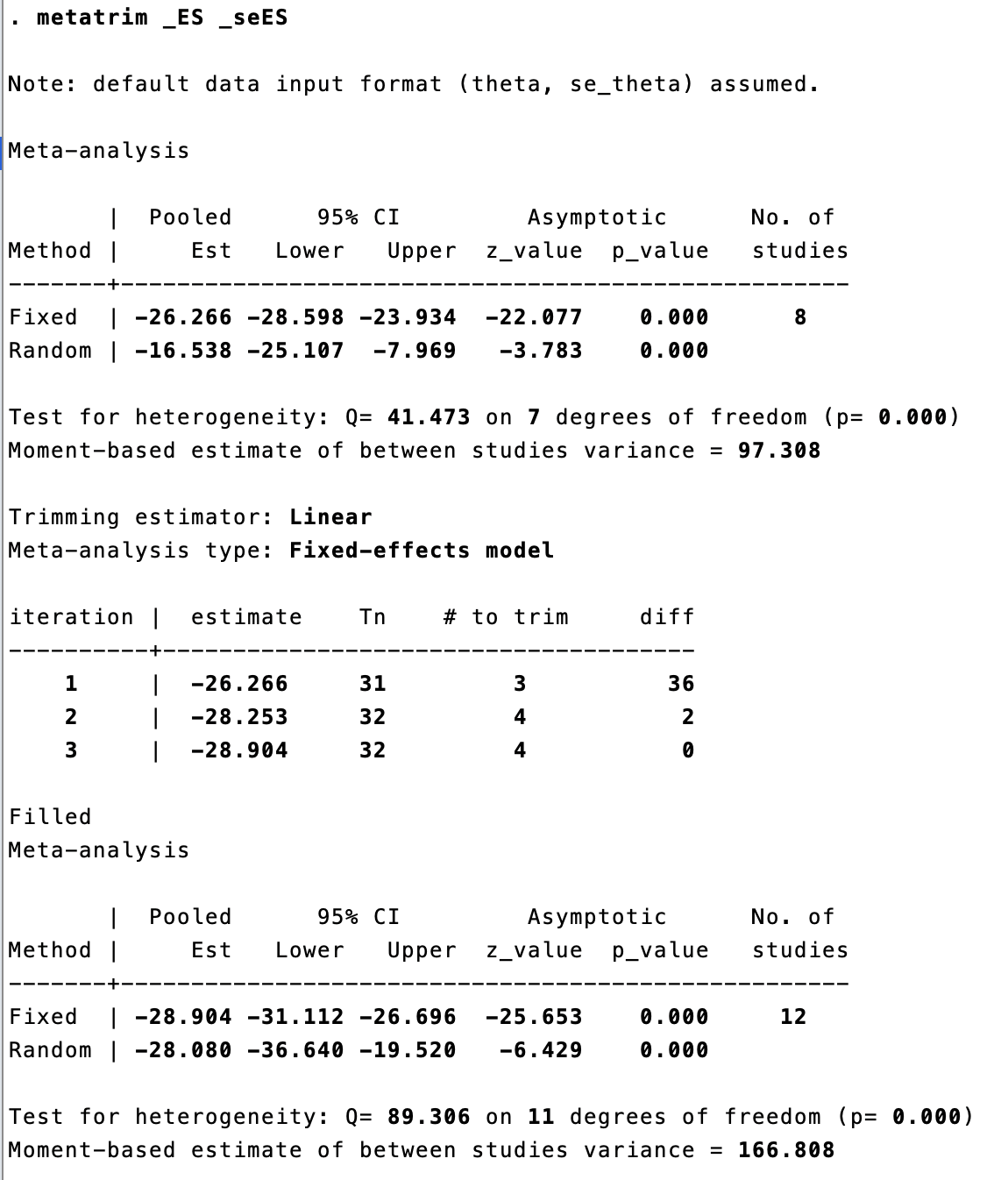


Figure S12 The trim-and-fill result of TG
